# Novel Nitro-Heteroaromatic Antimicrobial Agents for the Control and Eradication of Biofilm-Forming Bacteria

**DOI:** 10.3390/antibiotics10070855

**Published:** 2021-07-14

**Authors:** Heidi N. Koenig, Gregory M. Durling, Danica J. Walsh, Tom Livinghouse, Philip S. Stewart

**Affiliations:** 1 Department of Chemistry and Biochemistry, Montana State University, Bozeman, MT 59717, USA; heidi.koenig@montana.edu; 2 Department of Chemistry and Biochemistry, University of Notre Dame, Notre Dame, IN 46556, USA; gregory.durling@student.montana.edu (G.M.D.); dwalsh9@nd.edu (D.J.W.); 3 Center for Biofilm Engineering, Montana State University, Bozeman, MT 59717, USA; phil_s@montana.edu

**Keywords:** nitrothiazole, nitrobenzothiazole, nitrofuran, antimicrobials, biofilms, nosocomial infections

## Abstract

The synthesis and biological activity of several novel nitrothiazole, nitrobenzothiazole, and nitrofuran containing antimicrobial agents for the eradication of biofilm-forming Gram-negative and Gram-positive pathogens is described. Nitazoxanide (NTZ), nitrofurantoin, and furazolidone are commercial antimicrobials which were used as models to show how structural modification improved activity toward planktonic bacteria via minimum inhibitory concentration (MIC) assays and biofilms via minimum biofilm eradication concentration (MBEC) assays. Structure–activity relationship (SAR) studies illustrate the ways in which improvements have been made to the aforementioned antimicrobial agents. It is of particular interest in this regard that the introduction of a chloro substituent at the 5-position of NTZ (analog **1b**) resulted in marked activity enhancement, as did the replacement of the 2-acetoxy substituent in the latter compound with a basic amine group (analog **7b**). It is also of importance that analog **4a**, which is a simple methacrylamide, displayed noteworthy activity against *S. epidermidis* biofilms. These lead compounds identified to have high activity towards biofilms provide promise as starting points in future pro-drug studies.

## 1. Introduction

### 1.1. Biofilms in Nosocomial Infections

The emergence of multi-drug resistant bacteria has been largely attributed to the frequent use of antibacterial agents in the medical setting. These bacteria have developed several different mechanisms of resistance, e.g., efficient cellular efflux clearing, alterations to the target binding site, and biofilm formation. Bacteria that have the propensity to form biofilms have presented a considerable challenge for the medical field in the treatment of nosocomial infections. Biofilms are communities of bacteria that have adhered to a surface, either biotic or abiotic. Once attached, cells secrete extracellular polymeric substances (EPS) composed of extracellular DNA, polysaccharides, and proteins. The EPS traps nutrients and water within the biofilm, allowing cells to mature under nutrient rich conditions while being protected from desiccation, host immune defenses, and antimicrobial agents [1,2,3,4,5]. Biofilms are associated with three out of four microbial infections in the body and are responsible for around 1.7 million hospital-acquired infections per year [3], resulting in nearly 100 thousand deaths annually in the United States [4]. Bacteria deep within these films reside dormant, often allowing them to survive exposure to antibiotics or other bactericidal agents and regenerate the biofilm, making them more likely to achieve additional mechanisms of resistance.

The Center for Disease Control (CDC) estimates that as many as 2.8 million nosocomial infections occur annually in the United State, leading to 35,000 deaths and as much as $21–34 billion spent to treat these infections. Biofilms are implicated in the majority of these nosocomial infections [6]. Therefore, the development of drugs that are able to overcome this mechanism of resistance is of utmost importance and would have a profound impact in medicine.

### 1.2. Antimicrobial Design

Nitazoxanide is a broad-spectrum antiparasitic and broad-spectrum antiviral drug belonging to the thiazolide class. It is primarily used as a first-line treatment for infections caused by *Cryptosporidium parvum* and *Giardia lamblia* [7,8]. When nitazoxanide is metabolized, it is deacetylated to produce an active metabolite, tizoxanide [9]. Thiazolides interfere with the pyruvate:ferrodoxin oxidoreductase (PFOR) enzyme-dependent electron transfer reaction [10]. Nitazoxanide outcompetes the substrate pyruvate by targeting the vitamin cofactor thiamine pyrophosphate (TPP). Mechanistic studies suggest nitazoxanide is biologically active as an anion and abstracts a proton from the TPP:pyruvate intermediate—which ultimately halts the conversion of pyruvate to acetate, a key component for energy production. Docking studies have shown the nitro-group is necessary for nitazoxanide’s activity [11]. Nitazoxanide’s mode of action holds promise of future drug development, as it does not interact with the PFOR enzyme directly and is therefore less likely to promote mutation-based drug resistant strains. PFOR is also a unique mechanism to the target group of pathogens not present in humans, making it a prime candidate for therapeutics [12,13,14]. 

Several analogs of nitazoxanide have been synthesized and studied previously but were evaluated exclusively toward planktonic cells of *Heliobacter pylori, Campylocbacter jejuni, Clostridium difficile* as well as the protozoa *Giardia lamblia* and *Cryptosporidium parvum*. These analogs optimized nitazoxanide by replacing the nitro group with halogens as well as adding a number of other groups to the aromatic ring with fluoro, chloro, cyano, nitro, and methoxy substituents [15,16]. In these studies, it was shown that 5-chloro and 5-methoxy substituents markedly increased activity. Although there has been very limited research of nitazoxanide against biofilms, it has been shown to inhibit biofilm formation in *E. coli* and *S. epidermidis* and decrease the cell viability of *C. difficile* biofilms [17].

**Figure 1:**
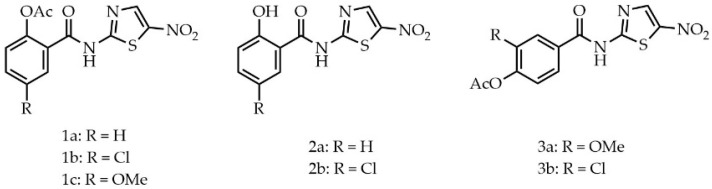
Nitazoxanide and initial analogs.

Several structurally differentiated nitazoxanide analogs herein were initially synthesized and biologically evaluated (Figure 1). Structure–activity relationship (SAR) studies are optimally conducted when direct comparisons can be made to a control molecule by introducing nominal changes, rather than altering many variables at once. With this goal in mind, substituents were introduced to the aryl ring to show how electron withdrawing groups (e.g., Cl) or electron donating groups (e.g., OCH_3_) can impact activity. Alternatively, the location of the nitrothiazolecarboxamide was shifted from the 2- to the 4-position relative to the acetoxy substituent.

**Figure 2:**
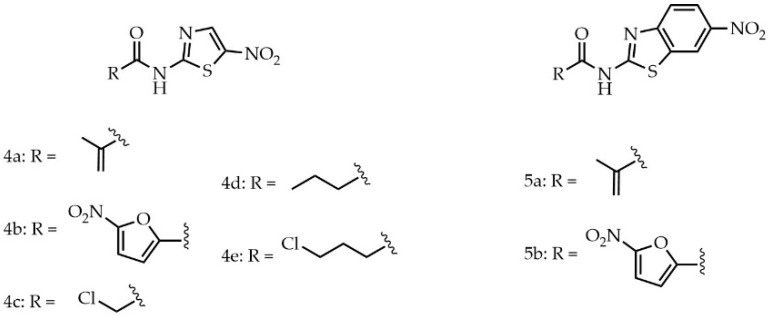
Second generation antimicrobials.

Further SAR analyses were conducted on the structurally simpler amides **4a**–**c** (Figure 2). The methacrylamide present in **4a** could potentially serve as a 1,4-nucleophile acceptor whereas the 2-chloroacetamide within **4c** and the 4-chlorobutyramide of **4e** could function as alkylating agents. As a control example, the simple butyramide **4d** was selected for comparison. In addition, the “dual-warhead” candidate **4b** was synthesized. A previous study has described dramatically enhanced activities of nitazoxanide analogs derived from 2-amino-6-nitrobenzothiazole [18]. Therefore, **5a** was prepared for a direct comparison to **4a** and the novel amide **5b** that also contains two nitroheteroaromatic antimicrobial “warheads” was synthesized and evaluated.

**Figure 3:**
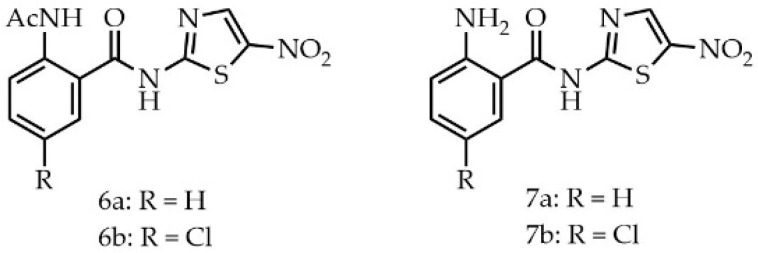
Analine and acetanilide analogs.

**Scheme 1:**
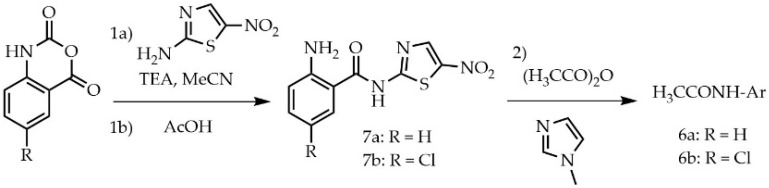
Synthesis of aniline and acetanilide analogs.

The acetoxy group is believed to be important for the activity of nitazoxanide (**1a**), since it can be hydrolyzed to the phenolic hydroxyl present in tizoxanide (**2a**) subsequent to cell penetration. As strategic alterations, anilines **7a**,**b** and acetanilides **6a**,**b** were synthesized and evaluated (Figure 3). The synthesis of these analogs was readily achieved by the acylation of 2-amino-5-nitrothiazole with the appropriate isatoic anhydride in MeCN using Et_3_N as the auxiliary base to provide **7a** and **7b** in 78% and 82% percent yields, respectively. Simple acetylation (Ac_2_O) then gave **6a**,**b** (Scheme 1).

**Figure 4:**
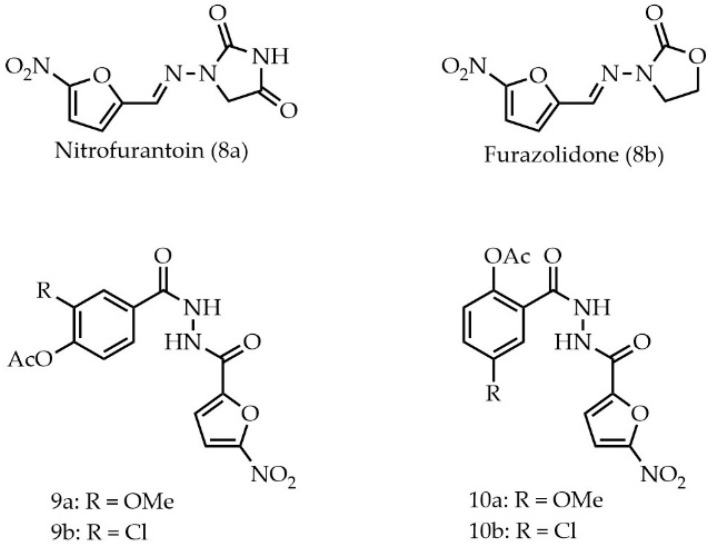
Nitrofuran analogs.

Other promising examples of drug models that include nitro functional groups are nitrofurantoin (**8a**) and furazolidone (**8b**). Both are nitrofurans, a class of drugs that have been shown to be active against both Gram-positive and Gram-negative pathogens, with the ability to inhibit mRNA and therefore interfere with gene expression [19]. Nitrofuranyl compounds have also been shown to be potent anti-tubercular agents [20,21,22] and nitrofuranyl benzimidazoles have shown great potency against methicillin-resistant *Staphylococcus aureus* and vancomycin-resistant *Enterococcus faecium* [23]. Nitrofurantoin is widely used to treat urinary tract infections. Mode of action studies show bacterial nitro reductases are responsible for converting nitrofurantoin into electrophilic intermediates which attack ribosomal proteins, effectively halting protein synthesis [24]. Furazolidone is used to treat gastrointestinal infections and is a monoamine oxidase inhibitor [25]. The 4-nitrofurancarboxylic hydrazides **9a**,**b** and **10** were readily prepared by the condensation of 4-nitrofuran carbohydrazide with the appropriate acyl chloride (Figure 4).

Investigation of these nitroheteroaromatic analogs as potential therapeutic antibiotics for the treatment of nosocomial biofilm infections is described. The aim was to find potent compounds with high activity against biofilms for potential starting points in further pro-drug studies.

## 2. Results and Discussion

### Biological Evaluation

Biological evaluation of the commercial antimicrobials and their nitroheteroaromatic analogs were conducted against *Staphylococcus epidermidis* and *Pseudomonas aeruginosa* in both the planktonic and biofilm states, via minimum inhibitory concentration (MIC) and minimum biofilm eradication concentration (MBEC) assays. The bacterium selected for this study were chosen based on comparison of Gram-negative and Gram-positive, as well as for their propensity to form biofilms. The Gram-negative bacterium selected for this study, *Pseudomonas aeruginosa,* is opportunistic, exhibits multi-drug resistance, and has a pronounced propensity to form biofilms. The Gram-positive bacterium selected for this study, *Staphylococcus epidermidis,* has the ability to form biofilms and has been implicated in chronic urinary tract infections, as well as persistent infections on medical devices [26,27,28]. *Staphylococcus epidermidis* strain ATCC 35984 was chosen as a representative Gram-positive organism with well-documented biofilm-forming ability. Although the primary focus of this study is biofilm eradication, MIC results involving *S. epidermidis* and *P. aeruginosa* in the planktonic state will be presented initially as background material. These results are assembled in Table 1.

Interestingly, nitazoxanide (**1a**) proved more active against both bacteria than its purported metabolite tizoxanide (**2a**). This contrast was even more substantial in the case of biofilms (*vide infra*). For the closely related analogs **1b** and **2b**, this trend was also observed with acetate **1b** being significantly more active toward *S. epidermidis,* but less so in the case of *P. aeruginosa*. For the positionally “inverted” acetate analog **3b**, comparatively excellent activity was observed against *S. epidermidis,* whereas **3a** was the most potent in the extended series toward *P. aeruginosa*.

For 2-amino-4-nitrothiazole (ANT) derivatives possessing potential nucleophile acceptors (e.g., **4a, 4c** and **4e**) no activity improvement was observed compared to the simple butyramide for *S. epidermidis*, but methacrylamide **4a** showed substantial activity against *P. aeruginosa*. Importantly, the “dual-warhead” candidate **5b** derived from 2-amino-6-nitrobenzothiazole proved highly effective against *S. epidermidis* in accord with the observations of Gua et al. [18]. It is also significant that anilines **7a** and **7b** showed high activity toward *S. epidermidis* with **7b** being noteworthy in this regard. Acetylation of the basic nitrogen in these compounds to give **6a** and **6b** resulted in a loss of activity, in sharp contrast to the corresponding to phenol **2b** vs. acetate **1b** (vide supra).

Within the 4-nitrofuran-2-carbohydrazide analog series excellent relative activities for **9b** and **10** were obtained against *S. epidermidis* with **10** also showing very good potency toward *P. aeruginosa*. It is of further interest that **10** corresponds structurally to **1b** in the “ANT” series, which proved outstanding in the MBEC studies that follow.

The unusual resilience of biofilm-mediated infections has been attributed to the retarded penetration of antimicrobial agents through binding to the robust EPS matrix, thereby hindering complete access throughout the biofilm [5,29,30,31,32]. Another more important obstacle in treating biofilms is the dormancy of cells within the interior, due to low levels of nutrients and oxygen. This reduced activity cell state is less susceptible to conventional antibiotics, which work most effectively on actively dividing cells [33,34]. Biofilms are therefore often many times more robust than planktonic bacteria toward antimicrobial agents.

The MBEC results that were obtained for *Staphylococcus epidermidis* and *Pseudomonas aeruginosa* are compiled in Table 2. It should first be emphasized that, once again, the presumed phenolic metabolites **2a** and **2b** were less potent that the corresponding acetates **1a** and **1b**, and in the case of tizoxanide (**2a**) this was significant. Furthermore, all of the ANT analogs exhibited substantially improved activities against *S. epidermidis* than nitazoxanide (**1a**). For the biofilms of current interest, acetate **1b** and aniline **7b** were noteworthy in respect of their potencies. In contrast to the findings of Ballard [15], the methoxy derivative **1c** was not particularly active. Methacrylamide **4a** also displayed high activity, and was comparable to **7b**, both being equipotent to nitazoxanide (**1a**) against *P. aeruginosa*. In contrast, the activities of the analogs derived from 2-amino-6-nitrobenzothiazole, **5a** and especially **5b**, were disappointing (see: MIC for **5b** above) and the ANT based “dual-warhead” analog **4b**. In the 4-nitrofuran-2-carbohydrazide analog series, only **9a** showed potential promise compared to the commercial antimicrobials.

## 3. Materials and Methods

### 3.1. Chemistry

All chemical reagents and conventional antibiotics (**1a**, **8a**, **8b**) were purchased from commercial sources (TCI America, Portland, OR, USA and Sigma Aldrich, Burlington, NC, USA) and used as received without further purification. Solvents utilized in filtrations, transfers, and chromatography were certified ACS grade. HEPES buffer and DE broth were purchased from Sigma Aldrich. Thin-layer chromatography was performed on Silicycle Glass Backed TLC Extra Hard Layer, 60 Å plates (thickness; 250 µm, indicator; F-254) and visualization was accomplished with UV light (254 nm), and/or potassium permanganate. All synthesized compounds were characterized (Figure S1) on a Bruker 500 MHz NMR Ascend Avance III HD (actively shielded) Spectrometer and a Bruker 600 MHz NMR Avance III NMR (actively shielded) Spectrometer. J values were depicted in hertz (Hz) and spin multiplicities were described as singlet (s), doublet (d), triplet (t), quartet (q), multiplet (m), and broad (b). Chemical shifts were reported in parts per million (δ), employing the solvent resonance as the internal standard.

#### 3.1.1. 2-Acetoxy-5-chloro-*N*-(5-nitrothiazol-2-yl)benzamide (**1b**)

A 10 mL round-bottom flask equipped with a magnetic stirring bar and poly-cap was charged with 2-acetoxy-5-chlorobenzoic acid (0.215 g, 1 mmol) and 3 mL of DCM. A catalytic amount of DMF was added. To this stirred solution, oxalyl chloride (0.174 mL, 2 mmol) was added. The reactant mixture was loosely capped and stirred for 15 min at room temperature. The corrosive by products were removed in vacuo before 2-amino-5-nitrothiazole (0.145 g, 1 mmol) was added and the flask was equipped with an ice bath. Anhydrous DCM (4 mL) was added, followed by the dropwise addition of triethyl amine (0.167 mL, 1.2 mmol). The reactant mixture was allowed to return to room temperature as it stirred overnight. The solvent was then removed in vacuo, the product mixture was diluted with EtOAc (5 mL) and successively extracted with water (2 × 5 mL) and brine (2 × 5 mL) to provide the crude product. Recrystallization from EtOAc provided the title compound (253 mg, 74%).

#### 3.1.2. 2-Acetoxy-5-methoxy-*N*-(5-nitrothiazol-2-yl)benzamide (**1c**)

A 10 mL round-bottomed flask equipped with a magnetic stirring bar and poly-cap was charged with 2-acetoxy-5-methoxybenzoic acid (0.210 g, 1 mmol) and 3 mL of DCM. A catalytic amount of DMF was added. To this stirred solution, oxalyl chloride (0.174 mL, 2 mmol) was added. The reactant mixture was loosely capped and stirred for 15 min at room temperature. The corrosive by products were removed in vacuo before 2-amino-5-nitrothiazole (0.145 g, 1 mmol) was added and the flask was equipped with an ice bath. Anhydrous DCM (4 mL) was added, followed by the dropwise addition of triethyl amine (0.167 mL, 1.2 mmol). The reactant mixture was allowed to return to room temperature as it stirred overnight. The solvent was then removed in vacuo, the product mixture was diluted with EtOAc (5 mL) and successively extracted with water (2 × 5 mL) to provide the crude product. Column chromatography using EtOAc:DCM (5:95) as the eluent (Rf: 0.24) provided the title compound (157 mg, 46%). ^1^H NMR (*d*_6_-DMSO, 500 MHz): *δ* 8.76 (1H, s), 7.48 (1H, s), 7.28 (1H, d, *J* = 1.4 Hz), 3.91 (3H, s), 2.28 (3H, s). ^13^C NMR (*d*_6_-DMSO, 500 MHz) *δ* 169.7, 156.8, 143.1, 142.5, 125.0, 119.6, 114.7, 56.36, 21.1.

#### 3.1.3. 2-Hydroxy-*N*-(5-nitrothiazol-2-yl)benzamide (**2a**)

A 10 mL round-bottomed flask equipped with a magnetic stirring bar and a yellow poly cap was charged with nitazoxanide (154 mg, 0.5 mmol), THF (5 mL) and hydrazine acetate (69 mg, 0.75 mmol). The reactant mixture was stirred for 20 h at room temperature as the product slowly precipitated. The THF was removed in vacuo and the remaining solid was suspended in 1:1 MeOH-H_2_O (5 mL). After stirring for 10 min at room temperature, the precipitate was collected by filtration and the filter cake was leached with 1:1 MeOH-H_2_O (2 × 3 mL). Drying in vacuo provided tizoxanide (124 mg, 94%). ^1^H NMR (*d*_6_-DMSO, 500 MHz): *δ* 12.20 (s, 1H), 7.90 (1H, dd, *J* = 1.6, 7.9 Hz), 7.49 (1H, ddd, *J* = 1.6, 7.0, 8.4 Hz), 7.04 (1H d *J* = 8.2 Hz), 6.99 (1H, dd, *J* = 7.3, 7.7 Hz), 3.59 (1H m), 3.54 (1H, s). ^13^C NMR (*d*_6_-DMSO, 500 MHz) *δ* 166.3, 162.4, 157.9, 143.0, 142.2, 135.4, 131.0, 120.2, 117.7, 117.2, 0.6.

#### 3.1.4. 5-Chloro-2-hydroxy-*N*-(5-nitrothizol-2-yl)benzamide (**2b**)

A 10 mL round-bottomed flask equipped with a magnetic stirring bar and a yellow poly cap was charged with **1b** (171 mg, 0.5 mmol), THF (5 mL) and hydrazine acetate (69 mg, 0.75 mmol). The reactant mixture was stirred for 20 h at room temperature as the product slowly precipitated. The THF was removed in vacuo and the remaining solid was suspended in 1:1 MeOH-H_2_O (5 mL). After stirring for 10 min at room temperature, the precipitate was collected by filtration and the filter cake was leached with 1:1 MeOH-H_2_O (2 × 3 mL). Drying in vacuo provided the title compound (139 mg, 93%).

#### 3.1.5. 2-Acetoxy-5-methoxy-*N*-(5-nitrothiazol-2-yl)benzamide (**3a**)

A 10 mL round-bottom flask equipped with a magnetic stirring bar and poly-cap was charged with 4-acetoxy-3-methoxybenzoic acid (0.210 g, 1 mmol) and 3 mL of DCM. A catalytic amount of DMF was added. To this stirred solution, oxalyl chloride (0.174 mL, 2 mmol) was added. The reactant mixture was loosely capped and stirred for 15 min at room temperature. The corrosive by products were removed in vacuo before 2-amino-5-nitrothiazole (0.145 g, 1.0 mmol) was added and the flask was equipped with an ice bath. Anhydrous DCM (4 mL) was added, followed by the dropwise addition of triethyl amine (0.167 mL, 1.2 mmol). The reactant mixture was allowed to return to room temperature as it stirred overnight. The solvent was then removed in vacuo, the product mixture was diluted with EtOAc (5 mL) and successively extracted with water (2 × 5 mL) and brine (2 × 5 mL) to provide the crude product. Recrystallization from EtOAc provided the title compound (196 mg, 58%). ^1^H NMR (*d*_6_-DMSO, 300 MHz): *δ* 8.70 (1H, s) 7.87 (1H, s), 7.74 (1H, m), 7.28 (1H, d, *J* = 12.7 Hz).

#### 3.1.6. 4-Acetoxy-3-chloro-*N*-(5-nitrothiazol-2-yl)benzamide (**3b**)

A 10 mL round-bottom flask equipped with a magnetic stirring bar and poly-cap was charged with 4-acetoxy-3-chlorobenzoic acid (0.215 g, 1 mmol) and 3 mL of DCM. A catalytic amount of DMF was added. To this stirred solution, oxalyl chloride (0.174 mL, 2 mmol) was added. The reactant mixture was loosely capped and stirred for 15 min at room temperature. The corrosive by products were removed in vacuo before 2-amino-5-nitrothiazole (0.145 g, 1.0 mmol) was added and the flask was equipped with an ice bath. Anhydrous DCM (4 mL) was added, followed by the dropwise addition of triethyl amine (0.167 mL, 1.2 mmol). The reactant mixture was allowed to return to room temperature as it stirred overnight. The solvent was then removed in vacuo, the product mixture was diluted with EtOAc (5 mL) and successively extracted with water (2 × 5 mL) and brine (2 × 5 mL) to provide the crude product. Recrystallization from EtOAc provided the title compound (215 mg, 63%). ^1^H NMR (*d*_6_-DMSO, 500 MHz): *δ* 13.69 (1H, s), 8.74 (1H, s), 8.37 (1H, d, *J* = 2.1 Hz), 8.14 (1H, dd, *J* = 2.1, 8.4 Hz), 7.57 (1H, d, *J* = 8.4 Hz), 2.39 (3H, s). ^13^C NMR (*d*_6_-DMSO, 500 MHz) *δ* 168.5. 150.7, 143.0, 130.8, 129.5, 126.9, 125.2, 20.9.

#### 3.1.7. *N*-(5-Nitrothiazol-2-yl)methacrylamide (**4a**)

A 10 mL round-bottomed flask equipped with a magnetic stirring bar and poly-cap was charged with 2-amino-6-nitrobenzothiazole (0.195 g, 1.00 mmol), THF (3 mL) and 1-methylimidazole (0.99 g, 1.2 mmol). The mixture was briefly warmed to dissolve the solid, whereupon it was cooled to 0 °C. Methacrylic anhydride (0.200 mL, 1.25 mmol) was then added with stirring and the reactant mixture was allowed to return to room temperature as it was stirred overnight. EtOAc (5 mL) was added and the mixture was successively extracted with water (2 × 5 mL) and brine (2 × 5 mL). Column chromatography using EtOAc:Hexanes (35:65) as the eluent (Rf: 0.22) provided the title compound (185 mg, 87%). ^1^H NMR (*d*_6_-DMSO, 500 MHz): *δ* 9.31 (1H, s), 8.35 (1H, s), 6.06 (1H, d, *J* = 0.52 Hz), 5.79 (1H, d, *J* = 1.53 Hz) 2.15 (3H, s). ^13^C NMR (*d*_6_-DMSO, 500 MHz) *δ* 165.3, 160.9, 140.7, 137.2, 124.5, 18.1, 1.02.

#### 3.1.8. 5-Nitro-*N*-(5-nitrothiazol-2-yl)furan-2-carboxamide (**4b**)

A 25 mL round-bottomed flask equipped with a magnetic stirring bar and a reflux condensor was charged with 4-nitrofuran-2-carboxylic acid (157 mg, 1.00 mmol), 1,2-dichloroethane (10 mL) and DMF (20 mg). The mixture was stirred at room temperature as oxalyl chloride (254 mg, 2.0 mmol) was slowly added in a dropwise fashion, with stirring, as HCl, CO and CO_2_ were evolved. After stirring for an additional 90 min at room temperature, the reactant mixture was concentrated in vacuo. The solid residue was dissolved in dry THF (10 mL) and transferred to a 50 mL flask, whereupon 2-amino-5-nitrothiazole (145 mg, 1.00 mmol) and 1-methylimidazole (99 mg, 1.2 mmol) were added with stirring at 0 °C. The resulting mixture was then stirred at room temperature for 24 h. Ethanol (25 mL was added and the precipitate was collected by filtration, followed by leaching of the filter cake with ethanol (2 × 5 mL) and drying to provide the product (227 mg, 80%). ^1^H NMR (*d*_6_-DMSO, 500 MHz): *δ* 8.76 (1H, s), 7.88 (1H, d, *J* = 3.8 Hz), 7.84 (1H, d, *J* = 3.9 Hz). ^13^C NMR (*d*_6_-DMSO, 500 MHz) *δ* 153.1, 142.4, 119.4, 113.7.

#### 3.1.9. 2-Chloro-*N*-(5-nitrothiazol-2-yl)acetamide (**4c**)

The title compound was prepared according to a literature procedure [35]. ^1^H NMR (D_6_-DMSO, 500 MHz): *δ* 13.45 (1H s), 8.66 (1H s), 4.51 (2H, s). ^13^C NMR (D_6_-DMSO, 500 MHz) *δ* 167.3, 161.8, 143.1, 142.7, 42.8.

#### 3.1.10. *N*-(5-nitrothiazol-2-yl)butyramide (**4d**)

A 10 mL round-bottomed flask equipped with a magnetic stirring bar and poly-cap was charged with 2-amino-5-nitrothiazole (0.145 g, 1.00 mmol), THF (3 mL) and 1-methylimidazole (0.99 g, 1.2 mmol). The mixture was briefly warmed to dissolve the solid, whereupon it was cooled to 0 °C. Butyric anhydride (204.5 µL, 1.25 mmol) was then added with stirring and the reactant mixture was stirred at room temperature overnight, then at 65 °C for 1.5 h. EtOAc (5 mL) was added and the mixture was successively extracted with water (2 × 3 mL) and brine (2 × 3 mL). Recrystallization from EtOAc provided the title compound (151 mg, 70%). ^1^H NMR (*d*_6_-DMSO, 500 MHz): *δ* 13.09 (1H, s), 8.68 (1H, s), 2.58 (2H, m) 1.70 (2H, m) 0.97 (3H, t, J = 7.4 Hz). ^13^C NMR (D_6_-DMSO, 500 MHz) *δ* 173.5, 162.2, 143.2, 142.1, 37.2, 18.3, 13.9.

#### 3.1.11. 4-chloro-*N*-(5-nitrothiazol-2-yl)butyramide (**4e**)

This compound was prepared similarly to **4d**. 1H NMR (D6-DMSO, 500 MHz): *δ* 8.63 (1H, s), 3.71 (2H, t, *J* = 6.5 Hz), 2.70 (2H, t, *J* = 7.3 Hz), 2.09 (2H, m). 13C NMR (D6-DMSO, 500 MHz) *δ* 172.7, 162.1, 143.1, 142.2, 45.1, 32.7, 27.5.

#### 3.1.12. *N*-(6-Nitrobenzo[d]thiazol-2-yl)methacrylamide (**5a**)

A 10 mL round-bottomed flask equipped with a magnetic stirring bar and poly-cap was charged with 2-amino-6-nitrobenzothiazole (0.195 g, 1.00 mmol), THF (3 mL), and 1-methylimidazole (0.99 g, 1.2 mmol). The mixture was briefly warmed to dissolve the solid, whereupon it was cooled to 0 °C. Methacrylic anhydride (0.200 mL, 1.25 mmol) was then added with stirring and the reactant mixture was allowed to return to room temperature as it was stirred overnight and then at 65 °C for 1.5 h. EtOAc (5 mL) was added and the mixture was successively extracted with water (2 × 5 mL) and brine (2 × 5 mL). Column chromatography using EtOAc:Hexanes (35:65) as the eluent (Rf: 0.32) provided the title compound (176 mg, 67%). ^1^H NMR (*d*_6_-DMSO, 500 MHz): *δ* 13.83 (1H, s), 9.11 (1H, d, *J* = 2.0 Hz), 8.34 (1H, dd, *J* = 2.4, 8.9 Hz) 7.94 (1H, m), 7.92 (1H, m), 7.86 (1H, d, *J* = 3.9 Hz). ^13^C NMR (*d*_6_-DMSO, 500 MHz) *δ* 153.1, 143.8, 122.6, 119.8, 119.2, 113.75.

#### 3.1.13. 5-Nitro-*N*-(6-nitrobenzo[d]thiazol-2-yl)furan-2-carboxamide (**5b**)

A 25 mL round-bottomed flask equipped with a magnetic stirring bar and a reflux condenser was charged with 4-nitrofuran-2-carboxylic acid (157 mg, 1.00 mmol), 1,2-dichloroethane (10 mL), and DMF (20 mg). The mixture was stirred at room temperature as oxalyl chloride (254 mg, 2.0 mmol) was slowly added in a dropwise fashion, with stirring, as HCl, CO and CO2 were evolved. After stirring for an additional 90 min at room temperature, the reactant mixture was concentrated in vacuo. The solid residue was dissolved in dry THF (5 + 5 mL) and transferred to a 50 mL flask, whereupon 2-amino-6-nitrobenzothiazole (195 mg, 1.00 mmol) and 1-methylimidazole (99 mg, 1.2 mmol) were added with stirring at 0 °C. The resulting mixture was then stirred at room temperature for 24 h. Ethanol (25 mL) was added and the precipitate was collected by filtration, followed by leaching of the filter cake with ethanol (2 × 5 mL) and drying to provide the title compound (247 mg, 74%). ^1^H NMR (*d*_6_-DMSO, 500 MHz): *δ* 12.83 (1H, s), 9.09 (1H, d, *J* = 2.4 Hz), 8.31 (1H, dd, *J* = 2.4 8.9 Hz), 7.91(1H, d, *J* = 8.9 Hz), 6.22 (1H s), 5.83 (1H, d, *J* = 1.4 Hz), 2.02 (1H, s). ^13^C NMR (*d*_6_-DMSO, 500 MHz) *δ* 125.2, 122.3, 120.9, 119.6, 18.7.

#### 3.1.14. 2-Acetamido-*N*-(5-nitrothizol-2-yl)benzamide (**6a**)

A 10 mL round-bottomed flask equipped with a magnetic stirring bar and poly-cap was charged with **7a** (1.00 mmol), DMAP (0.20 g) and dichloromethane (3 mL). Acetic anhydride (0.104 mL, 1.10 mmol) and triethylamine (0.160 mL, 1.15 mmol) were then sequentially added in a dropwise fashion with stirring. The reactant mixture was then stirred at room temperature overnight whereupon EtOAc (5 mL) was added and mixture was successively extracted with water (2 × 3 mL) and brine (2 × 3 mL) to provide the crude product. Column chromatography using EtOAc:DCM (25:75) as the eluent (Rf: 0.23) provided the title compound (273 mg, 89%). ^1^H NMR (*d*_6_-DMSO, 500 MHz): *δ* 13.48 (1H, s), 10.24 (1H, s), 8.69 (1H, s), 7.71 (2H, m), 7.57 (1H, ddd, *J* = 1.5, 6.8, 8.7 Hz), 7.26 (1H, ddd, *J* = 1.0, 7.4, 7.7 Hz), 2.03 (3H, s). ^13^C NMR (*d*_6_-DMSO, 500 MHz) *δ* 168.8, 132.9, 129.6, 124.2, 24.2.

#### 3.1.15. 2-Acetamido-5-chloro-*N*-(5-nitrothizol-2-yl)benzamide (**6b**)

A 10 mL round-bottomed flask equipped with a magnetic stirring bar and poly-cap was charged with **7b** (1.00 mmol), DMAP (0.20 g), and dichloromethane (3 mL). Acetic anhydride (0.104 mL, 1.10 mmol) and triethylamine (0.160 mL, 1.15 mmol) were then sequentially added in a dropwise fashion with stirring. The reactant mixture was then stirred at room temperature overnight whereupon EtOAc (5 mL) was added and mixture was successively extracted with water (2 × 3 mL) and brine (2 × 3 mL) to provide the crude product. Column chromatography using EtOAc:DCM (25:75) as the eluent (Rf: 0.26) provided the title compound (224 mg, 66%). ^1^H NMR (*d*_6_-DMSO, 500 MHz): *δ* 13.57 (1H, s), 10.44 (1H, s), 8.69 (1H, s), 7.82 (1H, m), 7.79 (1H, m), 7.61 (1H, dd, *J* = 2.3, 8.7 Hz), 2.04 (3H, s).

#### 3.1.16. 2-Amino-*N*-(5-nitrothiazol-2-yl)benzamide (**7a**)

A 10 mL round-bottomed flask equipped with a magnetic stirring bar and poly-cap was charged with (ANT) 2-amino-5-nitrothiazole (0.145 g, 1.0 mmol) and isatoic anhydride (0.163 g, 1.0 mmol). Anhydrous acetonitrile (3 mL) was then added and the reactant mixture was blanketed with nitrogen. Dry triethylamine (0.212 g, 154 µL, 2.1 mmol) was then added in one portion by gas-tight syringe, the poly-cap was tightly sealed, and the resulting mixture was vigorously stirred for 18 h. The reactant mixture was then quenched by the addition of acetic acid (0.120 g, 115 µL, 2.0 mmol, caution, gas evolution) with stirring and diluted with EtOAc (5 mL). The mixture was then filtered through silica gel (3 g) and concentrated in vacuo to provide the crude product. Column chromatography using EtOAc:DCM (5:95) as the eluent (Rf: 0.27) provided the title compound (182 mg, 69%). ^1^H NMR (*d*_6_-DMSO, 500 MHz): *δ* 8.89 (2H s), 8.78 (1H, s), 8.69 (1H, S), 7.89 (1H, d, *J* = 8.1 Hz), 7.29 (1H, ddd, *J* = 1.3, 6.8, 7.1 Hz) 6.82 (1H, d, *J* = 8.3 Hz) 6.58 (1H, ddd, *J* = 0.82, 7.1, 7.9 Hz). ^13^C NMR (*d*_6_-DMSO, 500 MHz) *δ* 115.17, 117.3, 129.8, 134.8, 151.9.

#### 3.1.17. 2-Amido-5-chloro-*N*-(5-nitrothizol-2-yl)benzamide (**7b**)

A 10 mL round-bottomed flask equipped with a magnetic stirring bar and poly-cap was charged with (ANT) 2-amino-5-nitrothiazole (0.145 g, 1.0 mmol) and 5-chloroisatoic anhydride (0.198 g, 1.0 mmol). Anhydrous acetonitrile (4mL) was then added and the reactant mixture was blanketed with nitrogen. Dry triethylamine (0.212 g, 154 µL, 2.1 mmol) was then added in one portion by gas-tight syringe, the poly-cap was tightly sealed, and the resulting mixture was vigorously stirred for 18 h. The reactant mixture was then quenched by the addition of acetic acid (0.120 g, 115 µL, 2.0 mmol, caution, gas evolution) with stirring and diluted with EtOAc (5 mL). The mixture was then filtered through silica gel (3 g) and concentrated in vacuo to provide the crude product. Column chromatography using EtOAc:DCM (5:95) as the eluent (Rf: 0.35) provided the title compound (215 mg, 72%). ^1^H NMR (*d*_6_-DMSO, 500 MHz): *δ* 8.89 (2H, s), 8.70 (1H, s), 7.91 (1H, dd, *J* = 2.3, 8.1 Hz), 7.29 (1H, m), 6.82 (1H, dd, *J* = 0.8, 8.5 Hz), 6.59 (1H, m). ^13^C NMR (*d*_6_-DMSO, 500 MHz) *δ* 163.2, 151.9, 134.8, 129.8, 117.4, 115.2, 110.4.

#### 3.1.18. 4-Acetoxy-3-methoxy-*N*-(5-nitrofuran-2-carbonyl)benzhydrazide (**9a**)

A 10 mL round-bottom flask equipped with a magnetic stirring bar and poly-cap was charged with 4-acetoxy-3-methoxybenzoic acid (0.2102 g, 1 mmol) and 3 mL of DCM. A catalytic amount of DMF was added. To this stirred solution, oxalyl chloride (0.174 mL, 2 mmol) was added. The reaction mixture was loosely capped and stirred for 15 min at room temperature. The corrosive by products were removed in vacuo before 5-nitrofuran-2-carbohydrazide (0.171 g, 1.0 mmol) was added and the flask was equipped with an ice bath. Anhydrous DCM (3.5 mL) was added, followed by the dropwise addition of triethyl amine (0.167 mL, 1.2 mmol). The reaction mixture was allowed to return to room temperature as it stirred overnight. The solvent was then removed in vacuo, the product mixture was diluted with EtOAc (5 mL) and successively extracted with water and brine to provide the crude product. Column chromatography using EtOAc:Hexanes (30:70) as the eluent (Rf: 0.27) provided the title compound (143 mg, 52%). ^1^H NMR (CDCl_3_, 500 MHz): *δ* 11.08 (1H, s), 10.75 (1H, s), 7.86 (1H, d, *J* = 3.91 Hz), 7.68 (1H, d, *J* = 1.84 Hz) 7.62 (1H, d, *J* = 3.91 Hz) 7.60 (1H, dd, *J* = 1.9, 8.2 Hz), 7.32 (1H, d, *J* = 8.1 Hz), 3.92(3H, s), 2.35 (3H, S). ^13^C NMR (CDCl_3_, 500 MHz) *δ* 167.6, 164.23, 155.14, 151.31, 150.2, 145.8, 141.6, 130.1, 122.4, 119.6, 115.9, 112.6, 111.1, 55.4, 19.7.

#### 3.1.19. 4-Acetoxy-3-chloro-*N*-(2-(5-nitrofuran-2-carbonyl)benzhydrazide (**9b**)

A 10 mL round-bottom flask equipped with a magnetic stirring bar and poly-cap was charged with 4-acetoxy-3-chlorobenzoic acid (0.215 g, 1 mmol) and 3 mL of DCM. A catalytic amount of DMF was added. To this stirred solution, oxalyl chloride (0.174 mL, 2 mmol) was added. The reaction mixture was loosely capped and stirred for 15 min at room temperature. The corrosive by products were removed in vacuo before 5-nitrofuran-2-carbohydrazide (0.171 g, 1.0 mmol) was added and the flask was equipped with an ice bath. Anhydrous DCM (4 mL) was added, followed by the dropwise addition of triethyl amine (0.167 mL, 1.2 mmol). The reaction mixture was allowed to return to room temperature as it stirred overnight. The solvent was then removed in vacuo. The product mixture was diluted with EtOAc (5 mL) and successively extracted with water and brine to provide the crude product. Column chromatography using EtOAc:Hexanes (80:20) as the eluent (Rf: 0.31) provided the crude product (132 mg, 49%). ^1^H NMR (*d*_6_-DMSO, 500 MHz): *δ* 11.05 (1H, s), 10.80 (1H, s), 8.08 (1H, d, *J* = 2.3 Hz), 7.91 (1H, dd, *J* = 2.5, 8.2 Hz), 7.78 (1H, d, *J* = 3.9 Hz), 7.55 (1H, d, *J* = 3.9 Hz), 7.50 (1H, d, *J* = 8.4 Hz), 2.36 (3H, s). ^13^C NMR (*d*_6_-DMSO, 500 MHz) *δ* 173.3 169.0, 160.9, 154.6, 151.6, 136.5, 131.6, 25.6.

#### 3.1.20. 2-Acetoxy-5-methoxy-*N*-(5-nitrofuran-2-carbonyl)benzhydrazide (**10a**)

A 10 mL round-bottom flask equipped with a magnetic stirring bar and poly-cap was charged with 2-acetoxy-5-methoxybenzoic acid (0.210 g, 1 mmol) and 3 mL of DCM. A catalytic amount of DMF was added. To this stirred solution, oxalyl chloride (0.174 mL, 2 mmol) was added. The reaction mixture was loosely capped and stirred for 15 min at room temperature. The corrosive by products were removed in vacuo before 5-nitrofuran-2-carbohydrazide (0.171 g, 1.0 mmol) was added and the flask was equipped with an ice bath. Anhydrous DCM (4 mL) was added, followed by the dropwise addition of triethyl amine (0.167 mL, 1.2 mmol). The reaction mixture was allowed to return to room temperature as it stirred overnight. The solvent was then removed in vacuo, the product mixture was diluted with EtOAc (5 mL) and successively extracted with water and brine to provide the crude product. Column chromatography using EtOAc:Hexanes (45:55) as the eluent (Rf: 0.32) provided the title compound (189 mg, 52%).

#### 3.1.21. 2-Acetoxy-5-chloro-*N*-(5-nitrofuran-2-carbonyl)benzhydrazide (**10b**)

A 10 mL round-bottom flask equipped with a magnetic stirring bar and poly-cap was charged with 2-acetoxy-5-chlorobenzoic acid (0.215 g, 1 mmol) and 3 mL of DCM. A catalytic amount of DMF was added. To this stirred solution, oxalyl chloride (0.174 mL, 2 mmol) was added. The reaction mixture was loosely capped and stirred for 15 min at room temperature. The corrosive by products were removed in vacuo before 5-nitrofuran-2-carbohydrazide (0.171 g, 1.0 mmol) was added and the flask was equipped with an ice bath. Anhydrous DCM (4 mL) was added, followed by the dropwise addition of triethyl amine (0.167 mL, 1.2 mmol). The reaction mixture was allowed to return to room temperature as it stirred overnight. The solvent was then removed in vacuo, the product mixture was diluted with EtOAc (5 mL) and successively extracted with water and brine to provide the crude product. Column chromatography using EtOAc:Hexanes (50:50) as the eluent (Rf: 0.28) provided the title compound (250 mg, 68%). ^1^H NMR (D_6_-DMSO, 500 MHz): *δ* 11.07 (1H, s), 10.59 (1H, s), 7.80 (1H, d, *J* = 3.9, 7.8 Hz), 7.69 (1H, dd, *J* = 2.6, 5.9 Hz), 7.67 (1H, dd, *J* = 2.4, 3.1 Hz), 7.55 (1H, d *J* = 3.9 Hz), 7.33 (1H, d, *J* = 8.5 Hz), 2.27 (3H, s), 3.41 (1H, s). ^13^C NMR (*d*_6_-DMSO, 500 MHz) *δ* 169.3, 163.6, 155.9, 152.4, 147.6, 146.7, 132.4, 130.5, 129.2, 129.1, 126.3, 117.2, 113.7, 21.2.

### 3.2. Microbiology

*P. aeruginosa* (PA01) and *S. epidermidis* (35984) were acquired from American Type Culture Collection (ATCC). All bacteria were sub-cultured onto tryptic soy agar (TSA) plates and incubated at 37 °C, 150 rpm for 24 h. Overnight cultures were prepared by transferring single colonies from the plates into 25 mL tryptic soy broth (TSB) in Erlenmeyer flasks and incubating at 37 °C, 150 rpm for 16 h. Then, 10 µL of overnight cultures were transferred into 50 mL of TSB, vortexed, and standardized to 10^6^–10^7^ CFU/mL via absorbance readings at 600 nm.

The minimum inhibitory concentrations (MICs) of all compounds against *S. epidermidis* and *P. aeruginosa* were determined using a modified 96-well plate assay previously described by Walsh et al. [26,27,28]. In short, Costar polystyrene 96-well plates were prepared with 150 µL of TSB and serial dilutions of the compound in dimethyl sulfoxide (DMSO). Both strains were cultured as described above in TSB and a replicator was used to inoculate each well. DMSO controls and blanks containing only TSB and inoculated cultures were also conducted. Plates were incubated at 37 °C for 24 h in static conditions. The optical density was taken at 600 nm via microplate reader at 0 h and at 24 h, and the difference measured to determine the MIC. Experiments were done in biological triplicate with technical duplicates. The same starting and final concentrations of each compound was used for each replicate, and for this reason we conclude no standard error.

The minimum biofilm eradication concentrations (MBECs) of all compounds against *S. epidermidis* and *P. aeruginosa* were determined as previously described by Walsh et al. [26,27,28] and as follows. Costar polystyrene 96-well plates were prepared with 150 µL of TSB. Both strains were cultured as described above in TSB and a replicator was used to inoculate each well. Plates were incubated at 37 °C for 24 h in static conditions. The planktonic phase cells and medium were gently removed and replaced with 150 µL of TSB and tenfold dilutions of compound being evaluated in DMSO. The 96-well plates were incubated for an additional 24 h at 37 °C. Wells were thoroughly agitated to ensure the biofilm was broken up from the surface of the wells using pipettes before three tenfold dilutions of each sample were taken and drop plated on TSA plates. TSA plates were incubated for 24 h at 37 °C. The minimum biofilm eradication concentration (MBEC) was determined to be the lowest concentration at which no bacterial growth occurred. The same starting concentration of each compound was used for each replicate and the same final concentration was observed for each trial, for this reason there is no standard error. Experiments were performed in biological triplicate with technical duplicates.

## 4. Conclusions

### 4.1. Key Findings

Structure–activity relationship (SAR) studies were conducted using a panel of 22 amide derivatives of 2-amino-5-nitrothiazole, 2-amino-6-nitrobenzothiazole, and 4-nitrofuran-2-carbazide against *Staphylococcus epidermidis* and *Pseudomonas aeruginosa* in both the planktonic and biofilm states. Significantly for biofilms, the introduction of a chloro substituent at the 5-position of NTZ (analog **1b**) results in excellent activity enhancement, as does the replacement of the 2-acetoxy substitutuent in **1b** with a basic amine group (analog **7b**). It is also of importance that analog **4a**, which is a simple methacrylamide, displays noteworthy activity against both *S. epidermidis* and *P. aeruginosa* biofilms.

### 4.2. Future Research

Many of the analogs described in this study showed significant increases in potency toward the Gram-positive pathogen *Staphylococcus epidermidis* in the biofilm state. The utilization of these against other Gram-positive biofilms implicated in nosocomial infections (e.g., *Staphylococcus aureus*, *Streptococcus pneumoniae*, and *Enterococcus faecalis*) will be a topic for future disclosures from these laboratories. Future evaluation of intrinsic toxicity is also needed since it is possible that the analogs that we have described with enhanced antibacterial efficacy are so because their general toxicity has increased. Additional studies involving iminodiacetate pro-drug conjugates [33] based on **1b**, **4a,** and **7b** are also forthcoming.

## Figures and Tables

**Table 1:** Minimum inhibitory concentrations (MICs, in mM) of synthesized nitrothiazolyl and nitrofuranyl containing analogs compared to the market-available drugs nitazoxanide, tizoxanide, nitrofurantoin, and furazolidone against nosocomial pathogens *Staphylococcus epidermidis* and *Pseudomonas aeruginosa*.

Compound	*S. epidermidis*	*P. aeruginosa*
Nitazoxanide (**1a**)	0.24	0.06
**1b**	0.019	1.9
**1c**	0.39	3.125
Tizoxanide (**2a**)	0.39	0.195
**2b**	3.9	7.8
**3a**	0.012	0.049
**3b**	0.006	0.195
**4a**	0.049	0.049
**4c**	0.012	0.39
**4d**	0.012	0.097
**4e**	0.098	3.125
**5a**	0.039	0.195
**5b**	<0.0015	0.098
**6a**	0.098	1.56
**6b**	0.78	1.56
**7a**	0.024	1.56
**7b**	0.003	0.78
Nitrofurantoin (**8a**)	0.049	0.098
Furazolidone (**8b**)	0.098	0.195
**9a**	0.39	0.78
**9b**	0.012	0.78
**10a**	0.0015	3.125
**10b**	0.006	0.195

**Table 2:** Minimum biofilm eradication concentrations (MBECs, in mM) of synthesized nitrothiazolyl and nitrofuranyl containing analogs compared to the market-available drugs nitazoxanide, tizoxanide, nitrofurantoin, and furazolidone against nosocomial pathogens *Staphylococcus epidermidis* and *Pseudomonas aeruginosa*.

Compound	*S. epidermidis*	*P. aeruginosa*
Nitazoxanide (**1a**)	50	3.125
**1b**	**0.16**	12.5
**1c**	1.56	25
Tizoxanide (**2a**)	>100	>100
**2b**	6.25	12.5
**3a**	1.56	25
**3b**	6.25	100
**4a**	1.56	3.125
**4b**	3.125	6.25
**4c**	3.125	50
**4d**	3.125	25
**4e**	12.5	50
**5a**	25	50
**5b**	3.125	25
**6a**	1.56	50
**6b**	1.56	25
**7a**	3.125	12.5
**7b**	**0.39**	3.125
Nitrofurantoin (**8a**)	3.125	12.5
Furazolidone (**8b**)	3.125	6.25
**9a**	3.125	12.5
**9b**	3.125	25
**10a**	12.5	25
**10b**	3.125	25

## Data Availability

Not applicable.

## Supplementary Materials

The following are available online at https://www.mdpi.com/article/10.3390/antibiotics10070855/s1, Figure S1: NMR Spectra.
