# Supplementary material for: Novel Nitro-Heteroaromatic Antimicrobial Agents for the Control and Eradication of Biofilm-Forming Bacteria
Source: Antibiotics (Basel). 2021 Jul 14;10(7):855. doi: 10.3390/antibiotics10070855 (PMC8300661; doi:10.3390/antibiotics10070855)
Supplement: Supplementary file 1 [file antibiotics-10-00855-s001.zip › antibiotics-1265159-supplementary.pdf]

antibiotics

MDPI

Article

Novel Nitro-Heteroaromatic Antimicrobial Agents for the Control and Eradication of Biofilm-Forming Bacteria

Heidi N. Koenig [1], Gregory M. Durling [2], Danica J. Walsh [2], Tom Livinghouse [1,*] and Philip S. Stewart [3]

[1] Department of Chemistry and Biochemistry, Montana State University, Bozeman, MT 59717, USA; heidi.koenig@montana.edu

[2] Department of Chemistry and Biochemistry, University of Notre Dame, Notre Dame, IN 46556, USA; gregory.durling@student.montana.edu (G.M.D.); dwalsh9@nd.edu (D.J.W.)

[3] Center for Biofilm Engineering, Montana State University, Bozeman, MT 59717, USA; phil_s@montana.edu

* Correspondence: livinghouse@chemistry.montana.edu; Tel.: +1-406-994-5408

Figure S1. NMR Spectra

1.1.1. 2-Acetoxy-5-Methoxy-N-(5-nitrothiazol-2-yl)benzamide (1c)

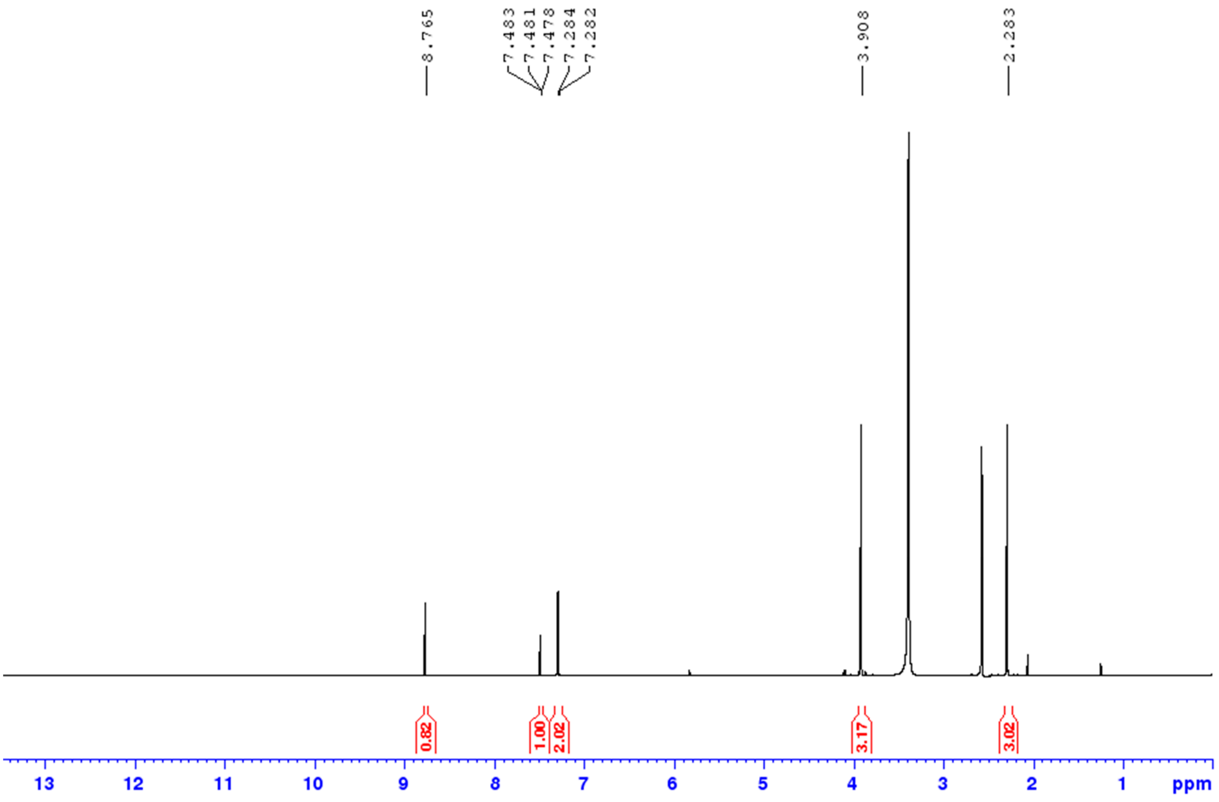

1H NMR (D6-DMSO, 500 MHz): 8.76 (1H, s), 7.48 (1H, m), 7.28 (1H, d, *J* = 1.4 Hz), 3.91 (3H, s), 2.28 (3H, s).

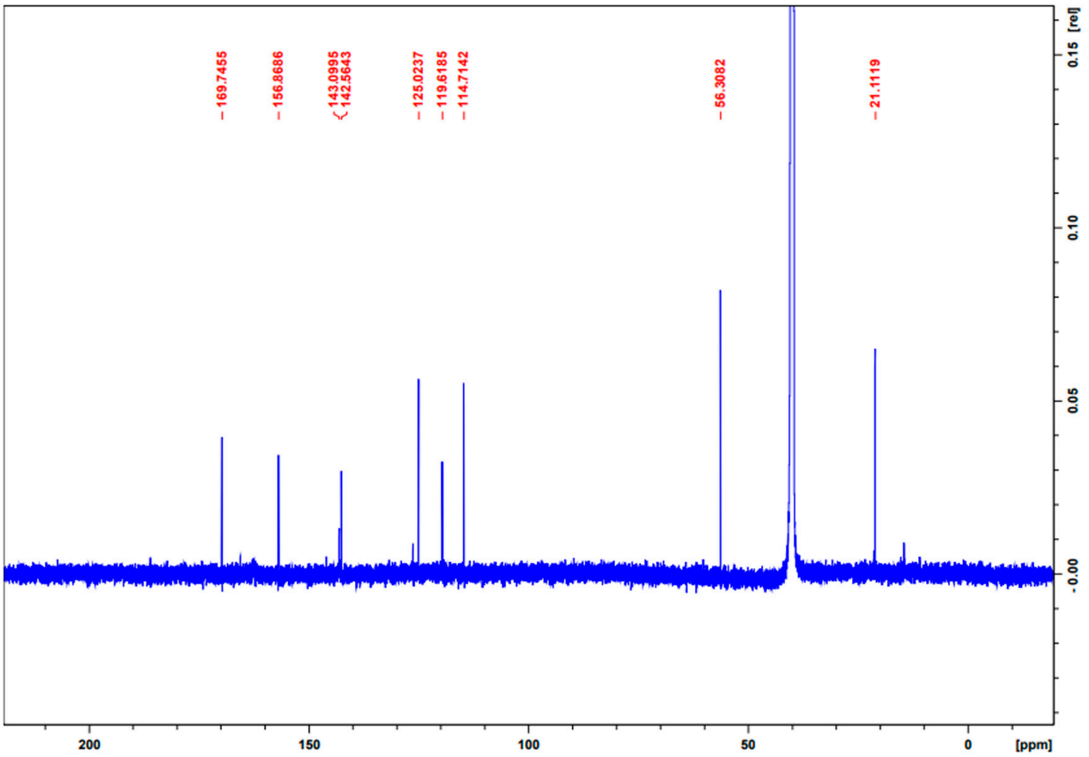

13C NMR (D6-DMSO, 500 MHz) 169.7, 156.8, 143.1, 142.5, 125.0, 119.6, 114.7, 56.36, 21.1.

1.1.2. 2-Hydroxy-N-(5-nitrothiazol-2-yl)benzamide (2a)

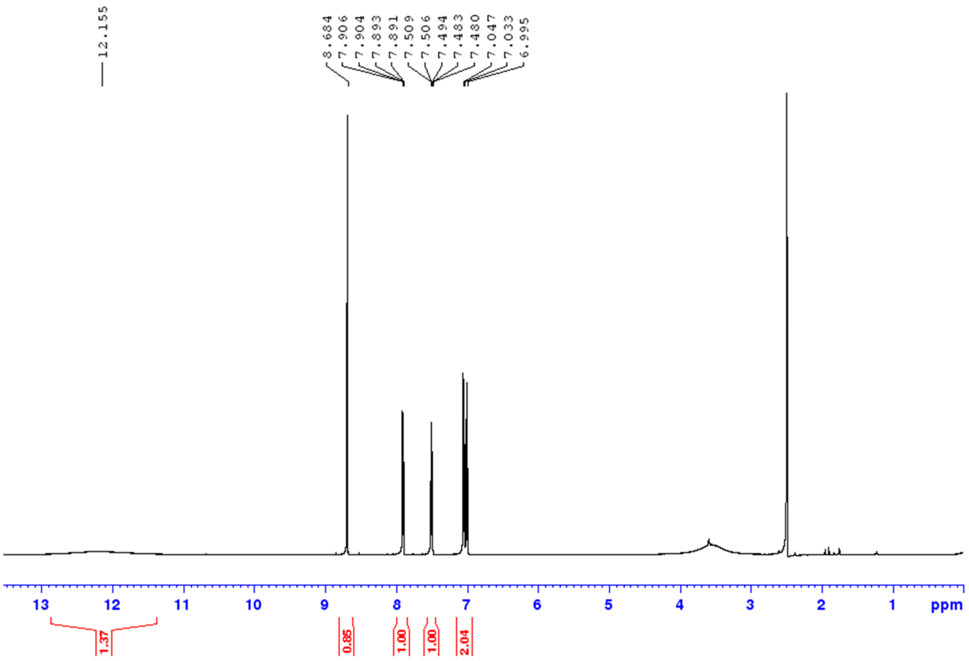

^{1}H NMR (D6-DMSO, 500 MHz): δ 12.20 (s, 1H), 8.68 (s, 1H), 7.90 (1H, dd, J = 1.6, 7.9 Hz), 7.49 (1H, ddd, J = 1.6, 7.0, 8.4 Hz), 7.04 (1H d J=8.2 Hz), 6.99 (1H, dd, J = 7.3, 7.7 Hz).

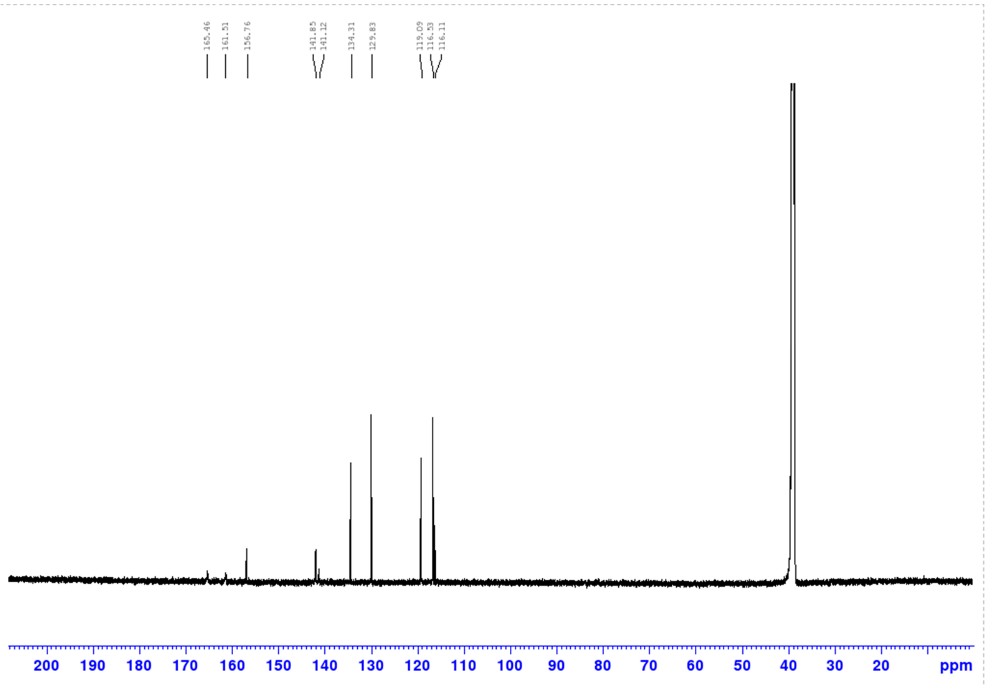

^{13}C NMR (D6-DMSO, 500 MHz) δ 166.3, 162.4, 157.9, 143.0, 142.2, 135.4, 131.0, 120.2, 117.7, 117.2.

1.1.3. 2-Acetoxy-5-methoxy-N-(5-nitrothiazol-2-yl)benzamide (3a)

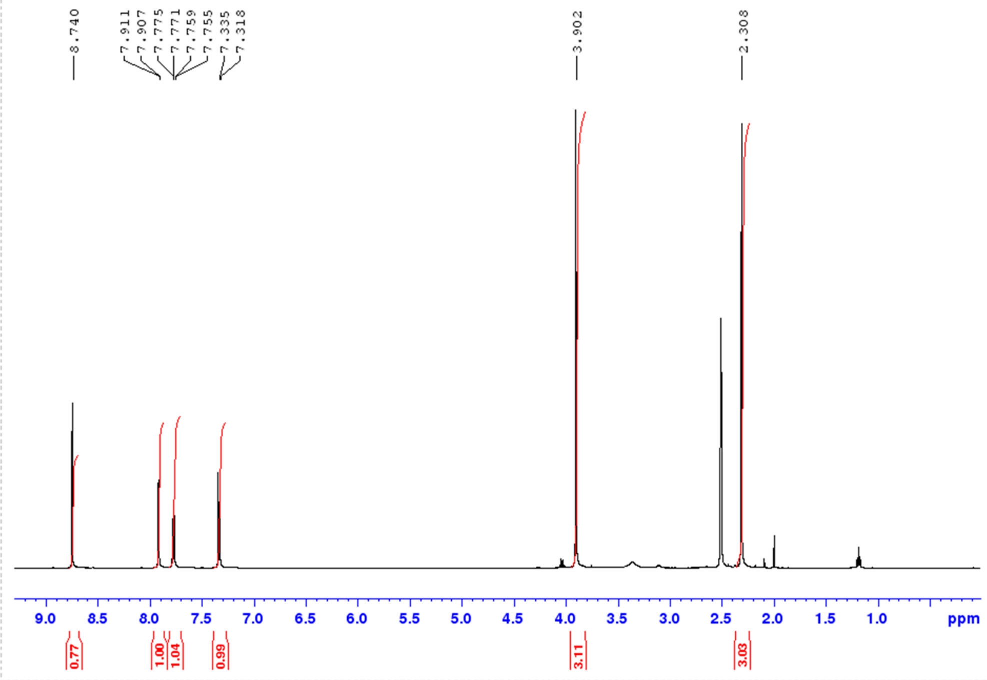

^{1}H NMR (D6-DMSO, 300 MHz): δ 8.74 (1H, s) 7.91 (1H, s), 7.76 (1H, m), 7.31 (1H, d, J = 12.7 Hz), 3.90 (3H, s), 2.31 (3H, s).

1.1.4. 4-Acetoxy-3-Chloro-N-(5-nitrothiazol-2-yl)benzamide (3b)

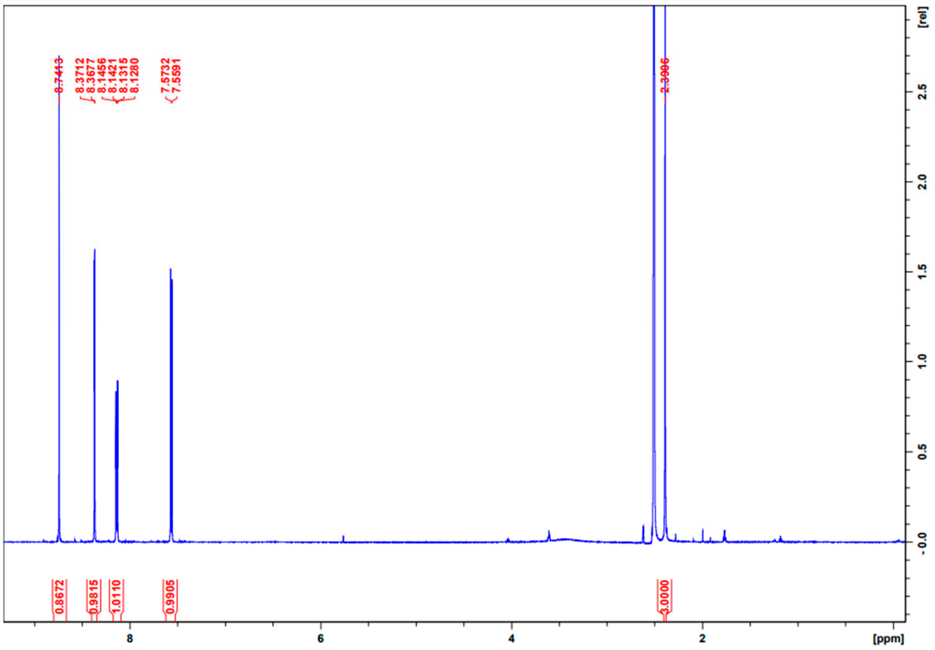

^{1}H NMR (D$_6$-DMSO, 500 MHz): δ 8.74 (1H, s), 8.37 (1H, d, J = 2.1 Hz), 8.14 (1H, dd, J = 2.1, 8.4 Hz), 7.57 (1H, d, J = 8.4 Hz), 2.39 (3H, s).

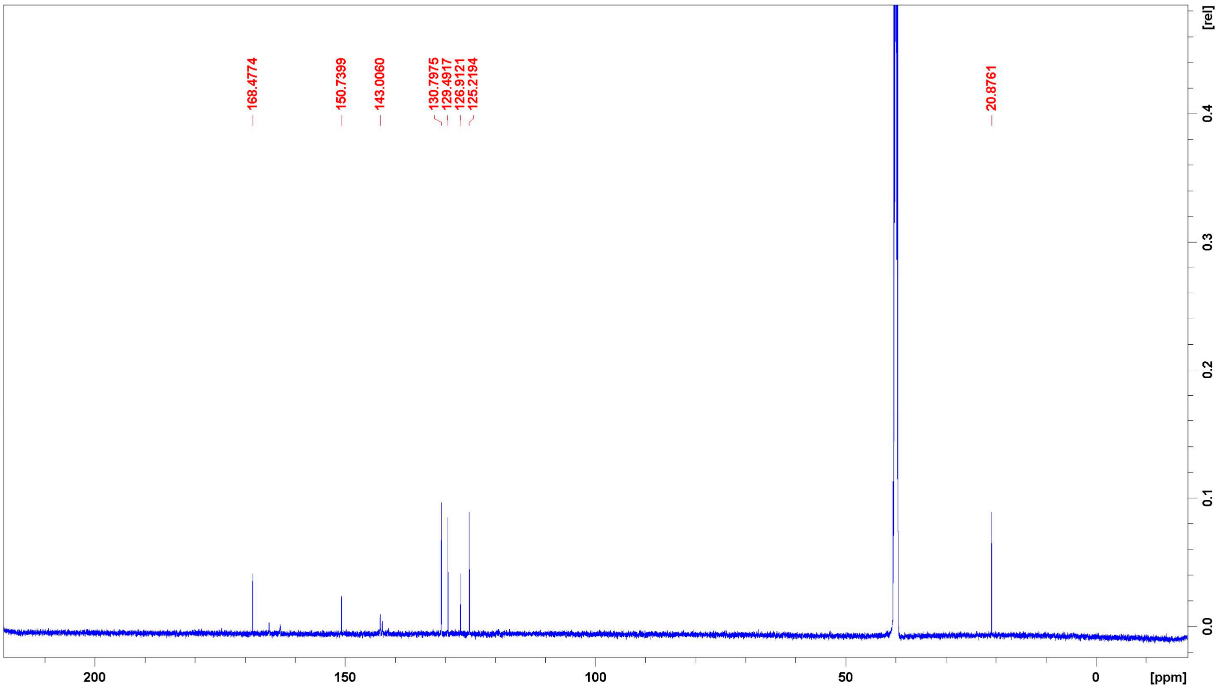

^{13}C NMR (D_6-DMSO, 500 MHz) δ 168.5. 150.7, 143.0, 130.8, 129.5, 126.9, 125.2, 20.9.

1.1.5. N-(5-Nitrothiazol-2-yl)methacrylamide (4a)

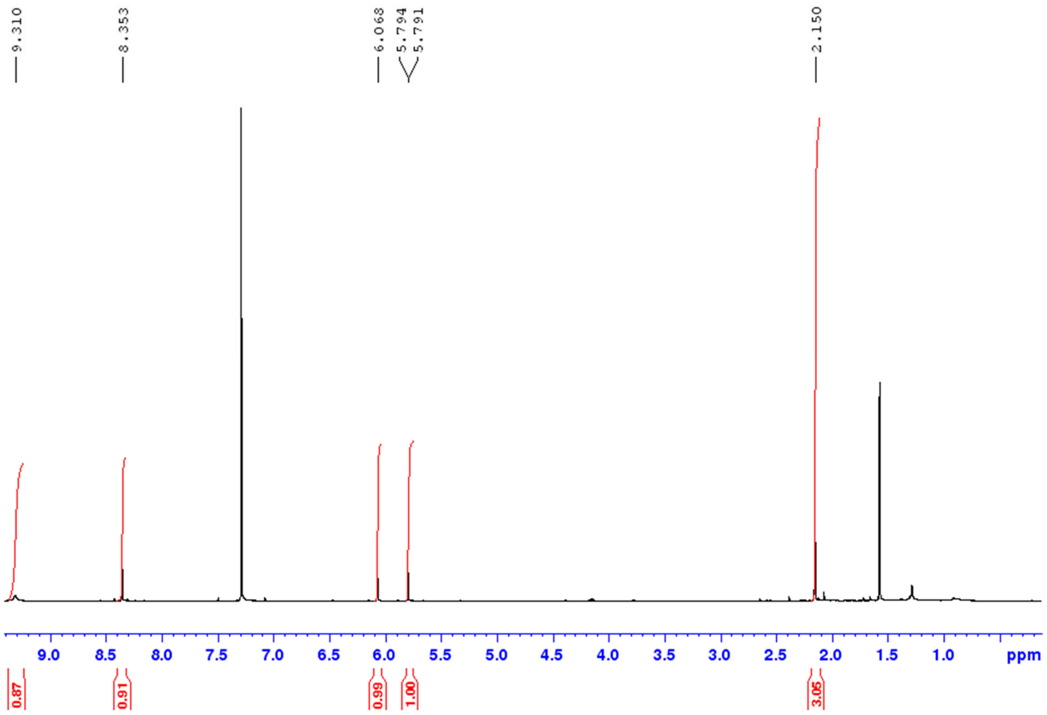

^{1}H NMR (D_6-DMSO, 500 MHz): δ 9.31 (1H, s), 8.35 (1H, s), 6.06 (1H, d, J = 0.52 Hz), 5.79 (1H, d, J = 1.53 Hz) 2.15 (3H, s).

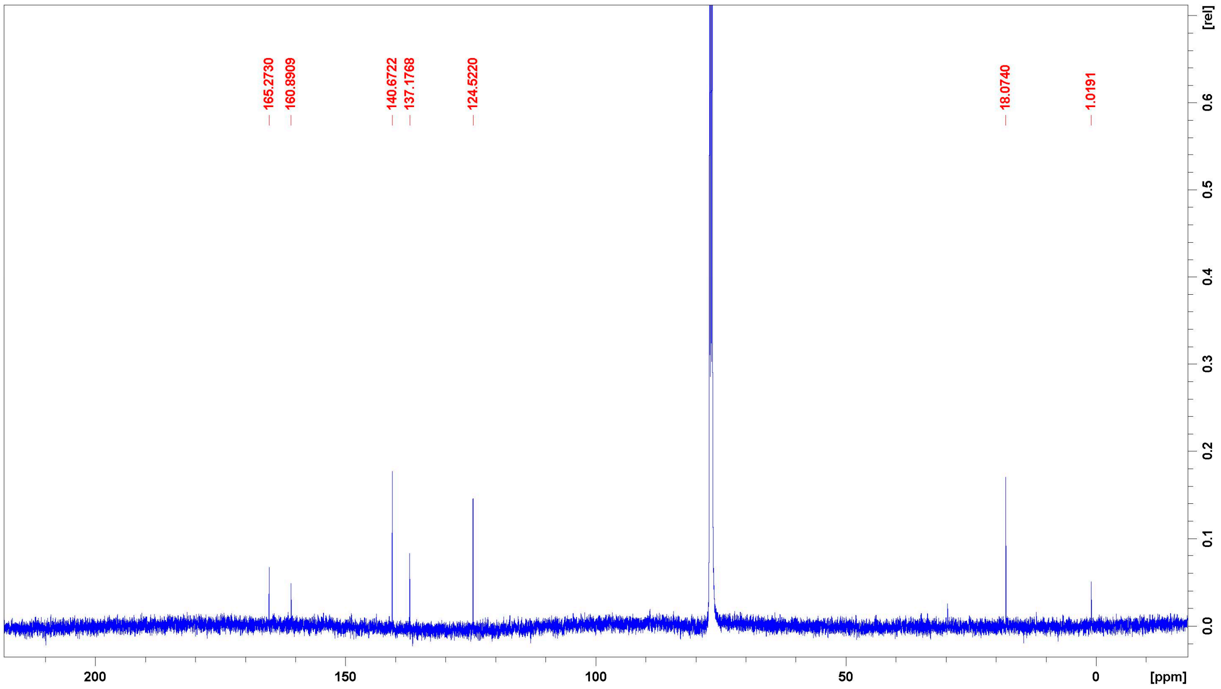

^{13}C NMR (D_6-DMSO, 500 MHz) δ 165.3, 160.9, 140.7, 137.2, 124.5, 18.1, 1.02.

1.1.6. 5-Nitro-N-(5-nitrothiazol-2-yl)furan-2-carboxamide (4b)

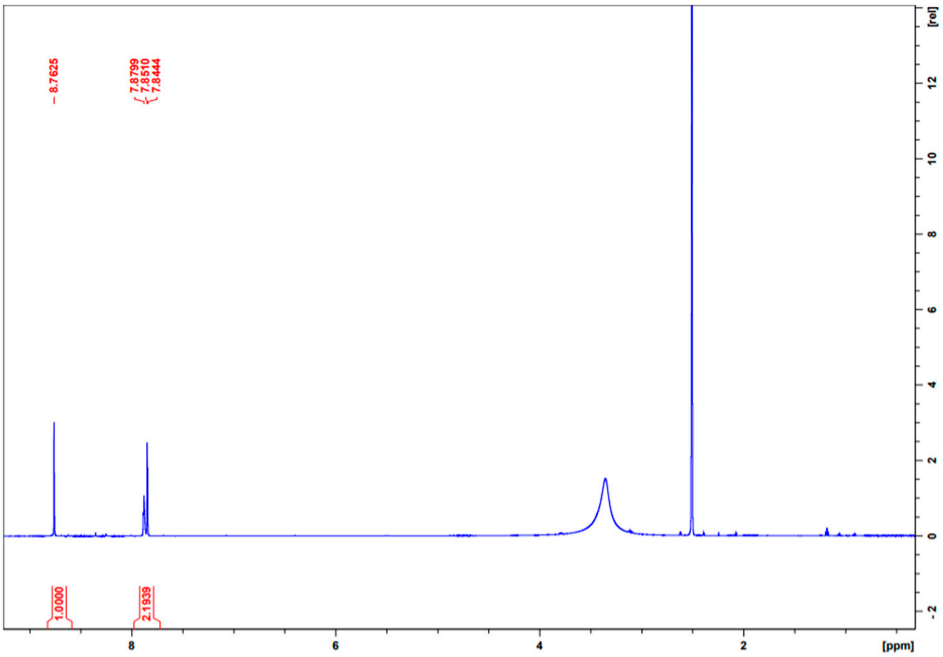

^{1}H NMR (D_6-DMSO, 500 MHz): δ 8.76 (1H, s), 7.88 (1H, d, J = 3.8 Hz), 7.84 (1H, d, J = 3.9 Hz).

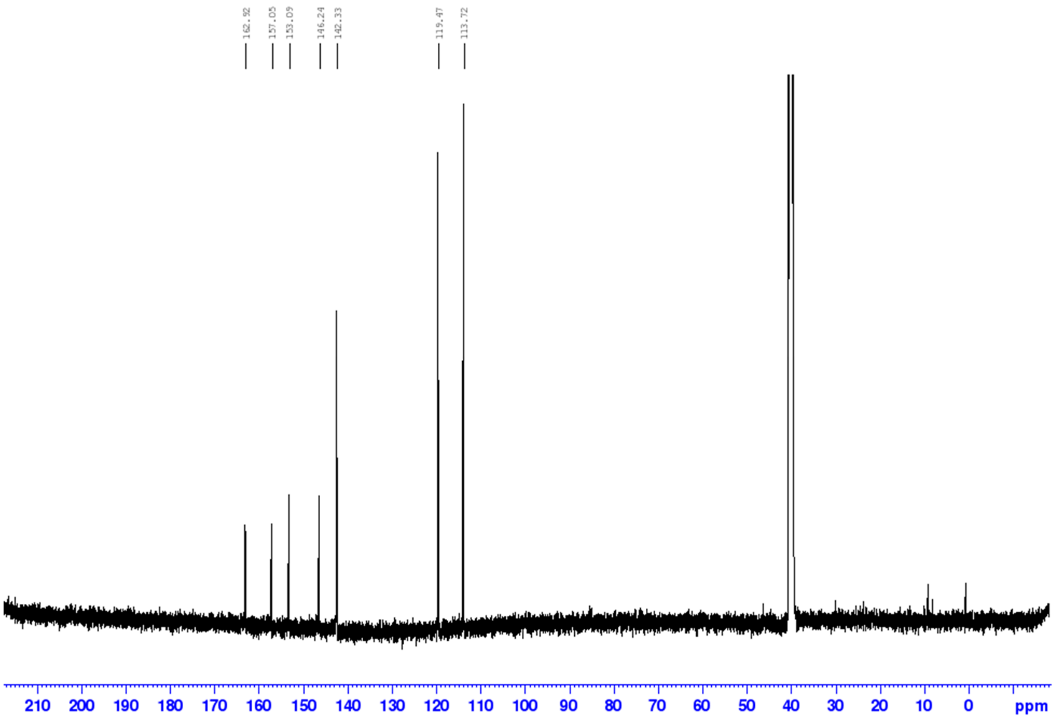

^{13}C NMR (D_6-DMSO, 500 MHz) δ 162.9, 157.1, 153.1, 146.2, 142.3, 119.5, 113.7.

1.1.7. 2-Chloro-N-(5-nitrothiazol-2-yl)acetamide (4c)

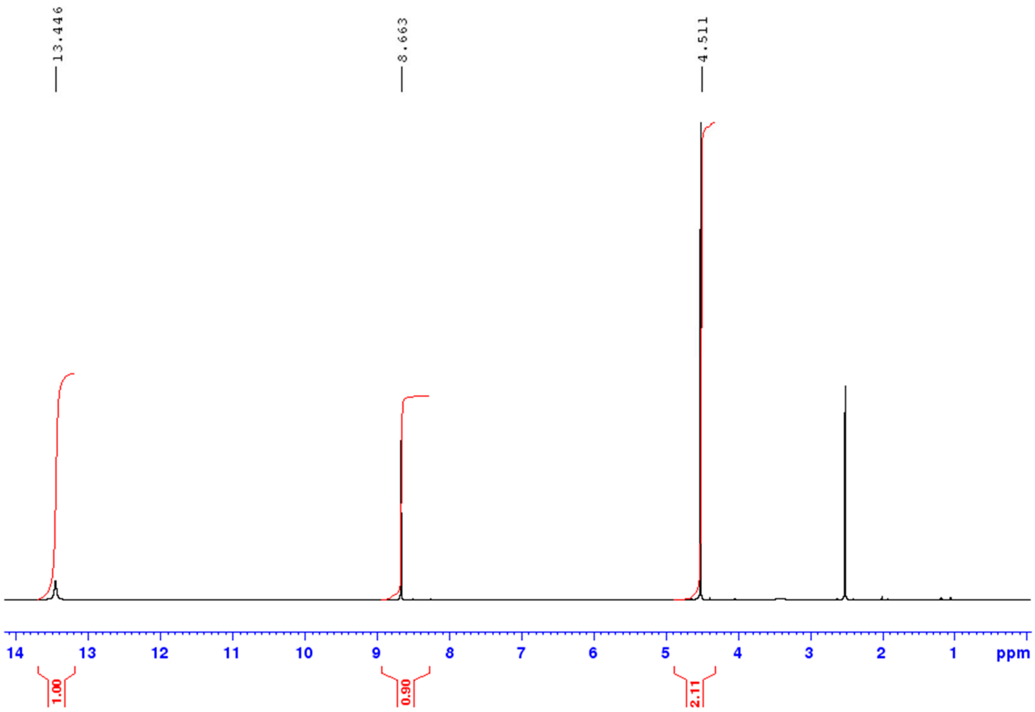

^{1}H NMR (D_6-DMSO, 500 MHz): δ 13.45 (1H s), 8.66 (1H s), 4.51 (2H, s).

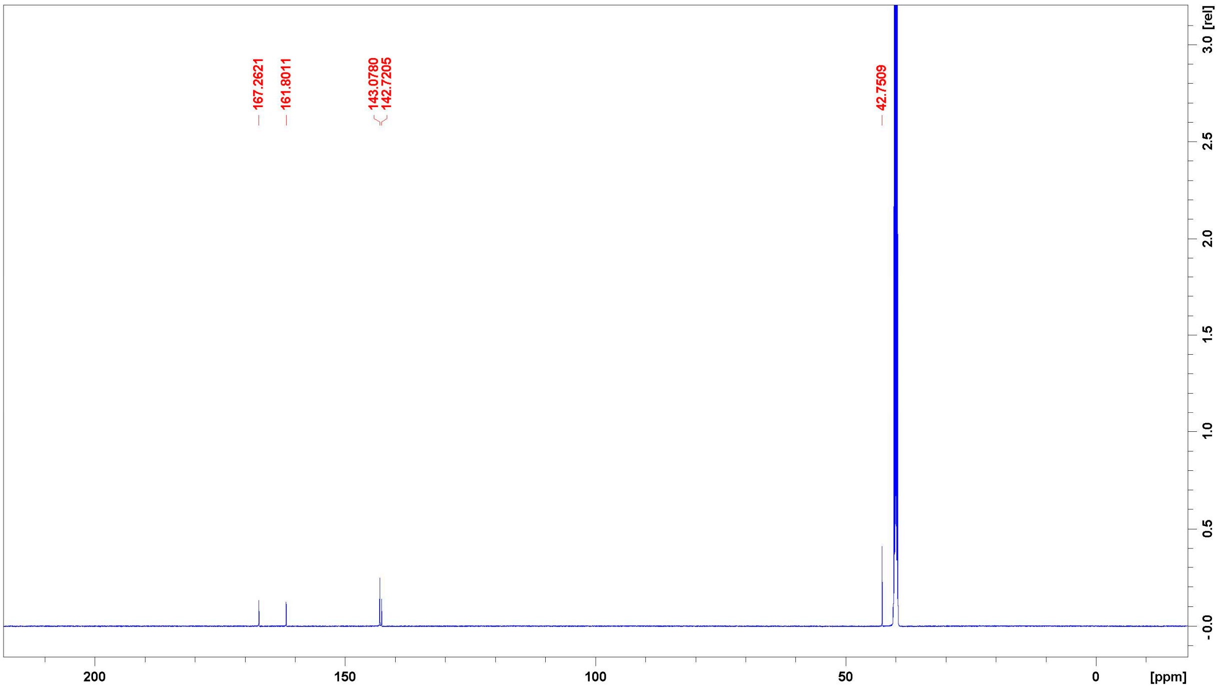

^{13}C NMR (D_6-DMSO, 500 MHz) δ 167.3, 161.8, 143.1, 142.7, 42.8.

1.1.8. N-(5-Nitrothiazol-2-yl)butyramide (4d)

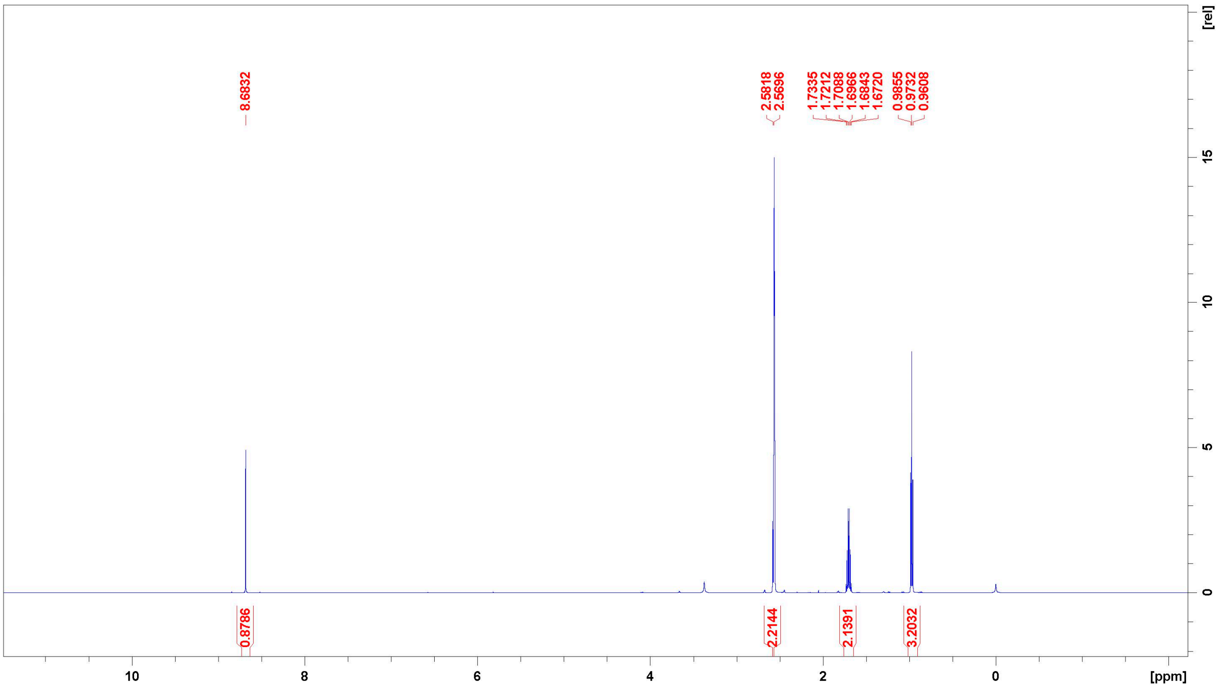

^{1}H NMR (D_6-DMSO, 500 MHz): δ 8.68 (1H, s), 2.58 (2H, m) 1.70 (2H, m) 0.97 (3H, t, *J* = 7.4 Hz).

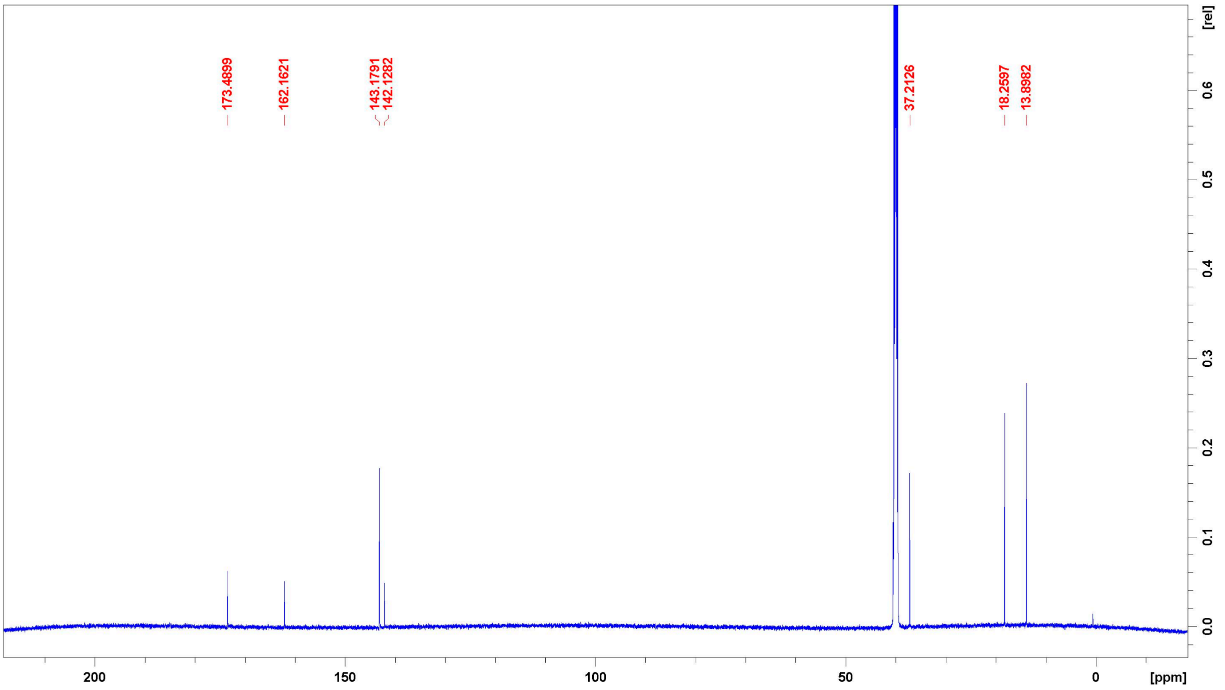

^{13}C NMR (D$_6$-DMSO, 500 MHz) δ 173.5, 162.2, 143.2, 142.1, 37.2, 18.3, 13.9.

1.1.9. 4-chloro-N-(5-nitrothiazol-2-yl)butyramide (4e)

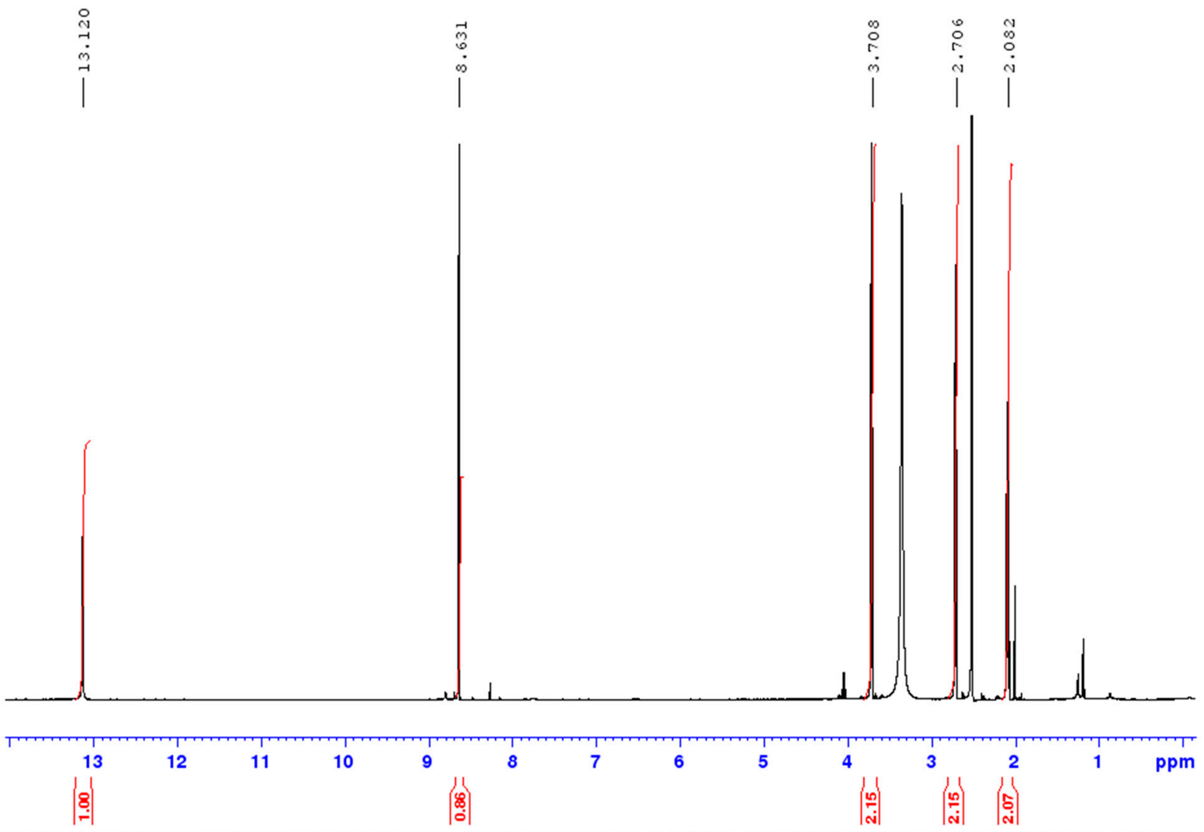

1H NMR (D6-DMSO, 500 MHz): 13.12 (1H, s), 8.63 (1H, s), 3.71 (2H, t, *J* = 6.5 Hz), 2.71 (2H, t, *J* = 7.3 Hz), 2.08 (2H, m).

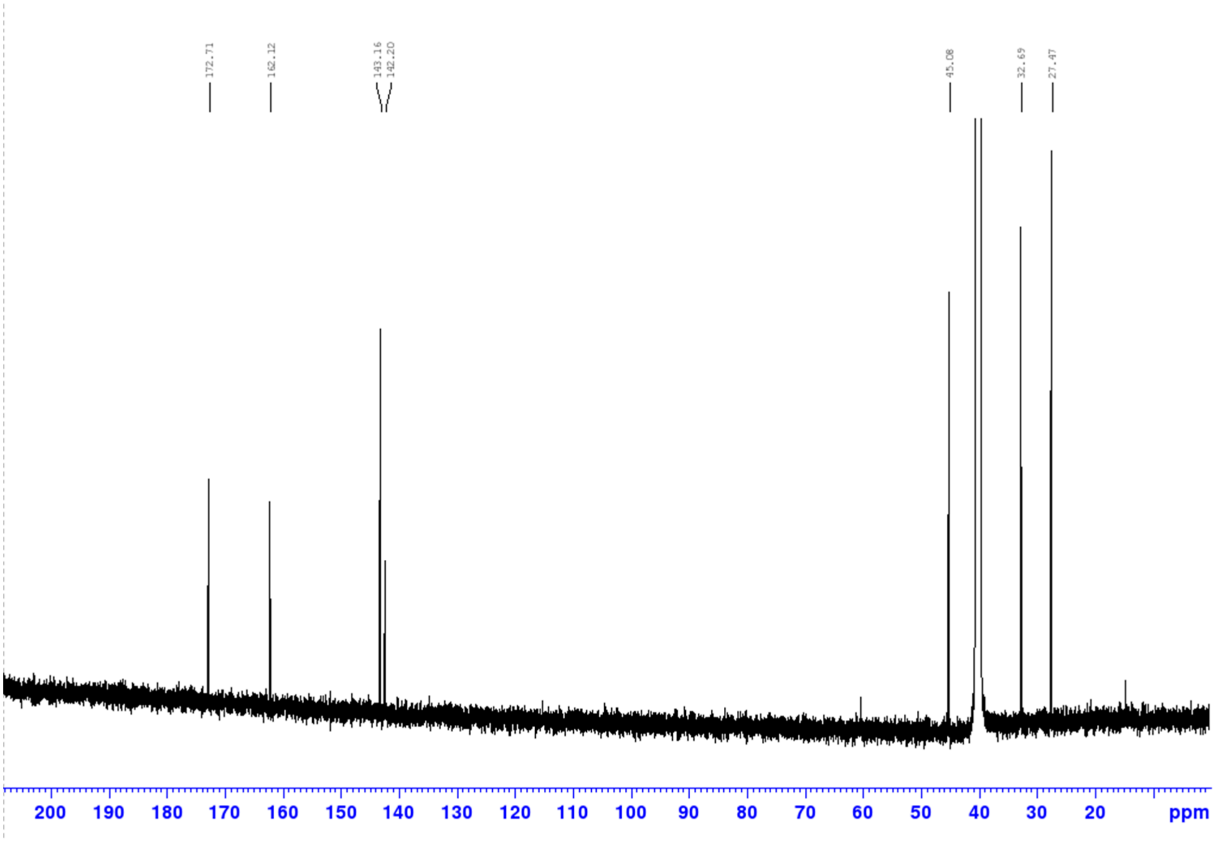

13C NMR (D6-DMSO, 500 MHz) 172.7, 162.1, 143.1, 142.2, 45.1, 32.7, 27.5.

1.1.10. N-(6-Nitrobenzo[d]thiazol-2-yl)methacrylamide (5a)

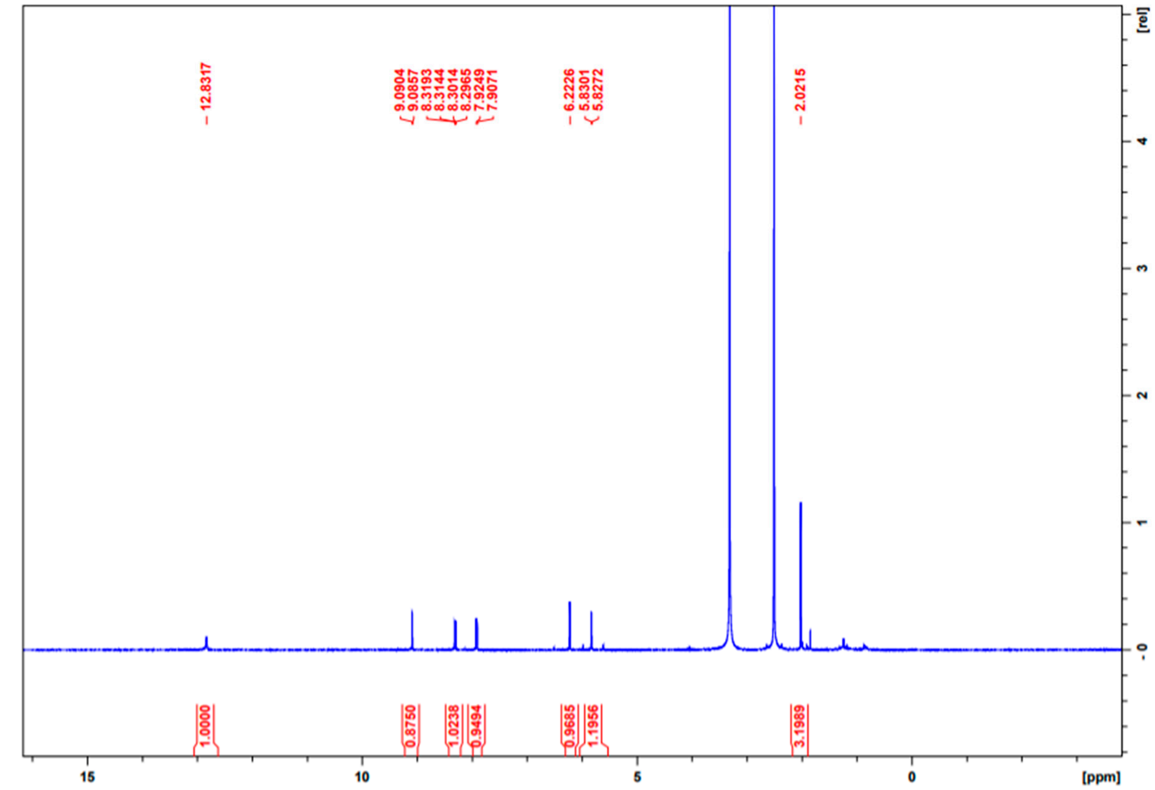

^{1}H NMR (D_6-DMSO, 500 MHz): δ 12.83 (1H, s), 9.11 (1H, d, J = 2.0 Hz), 8.34 (1H, dd, J = 2.4, 8.9 Hz), 7.92 (1H, d, J = 3.9 Hz) 6.22 (1H, s), 5.83 (1H, s), 2.02 (3H, s).

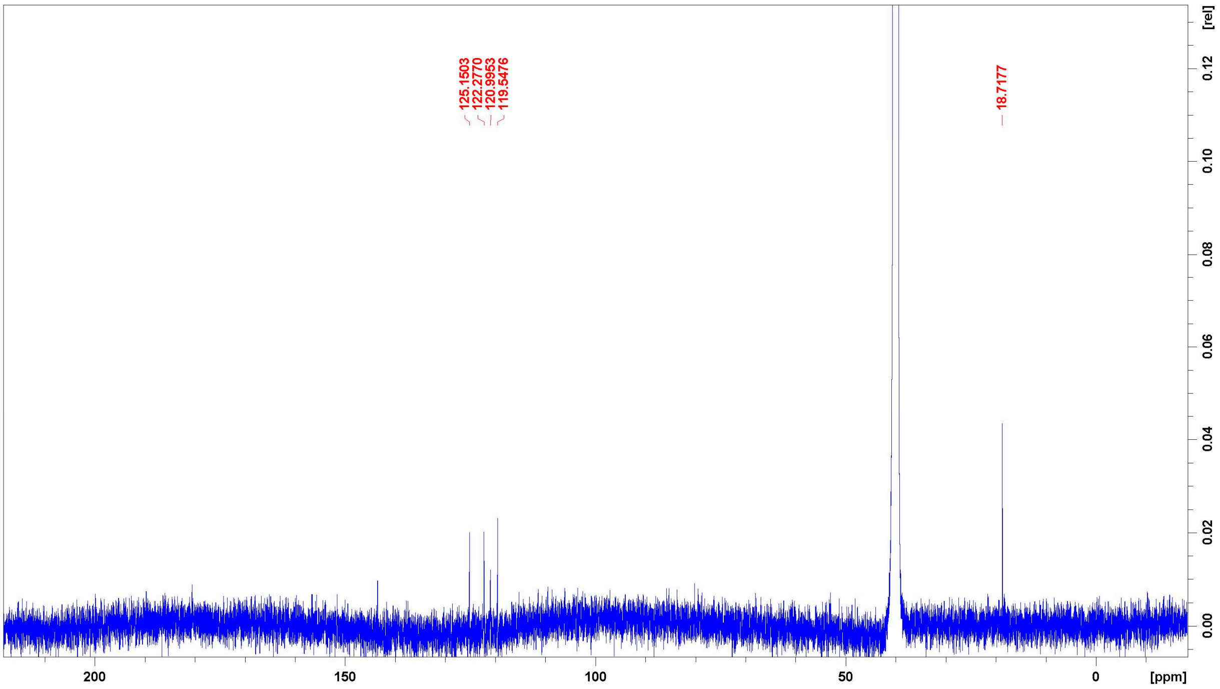

^{13}C NMR (D_6-DMSO, 500 MHz) δ 125.2, 122.3, 120.9, 119.5, 18.7.

1.1.11. 5-Nitro-N-(6-nitrobenzo[d]thiazol-2-yl)furan-2-carboxamide (5b)

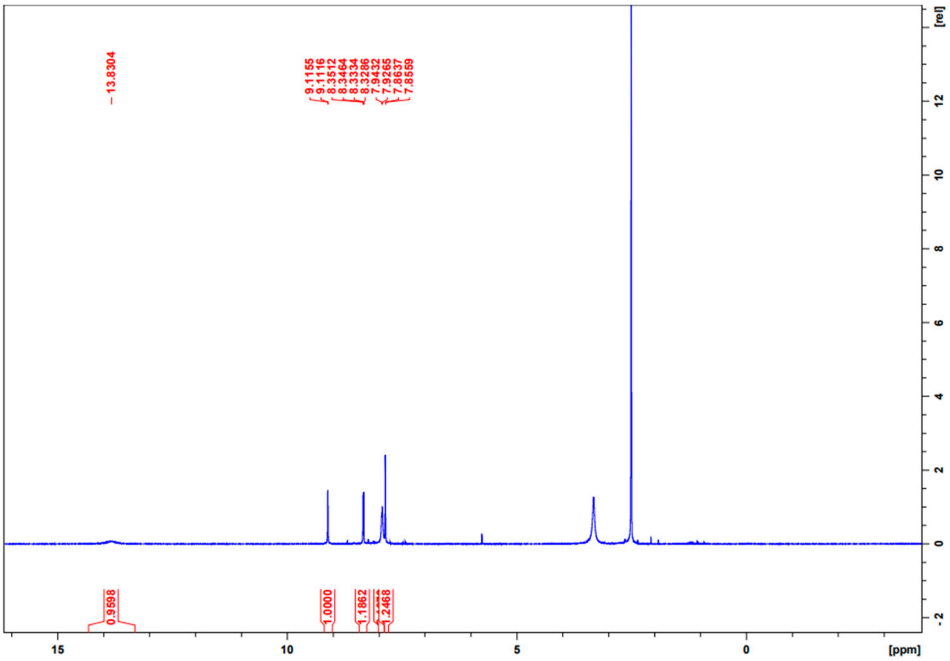

^{1}H NMR (D_6-DMSO, 500 MHz): δ 13.83 (1H, s), 9.12 (1H, d, J = 2.4 Hz), 8.33 (1H, dd, J = 2.4 8.9 Hz), 7.91(1H, d, J = 8.9 Hz), 7.86 (1H s).

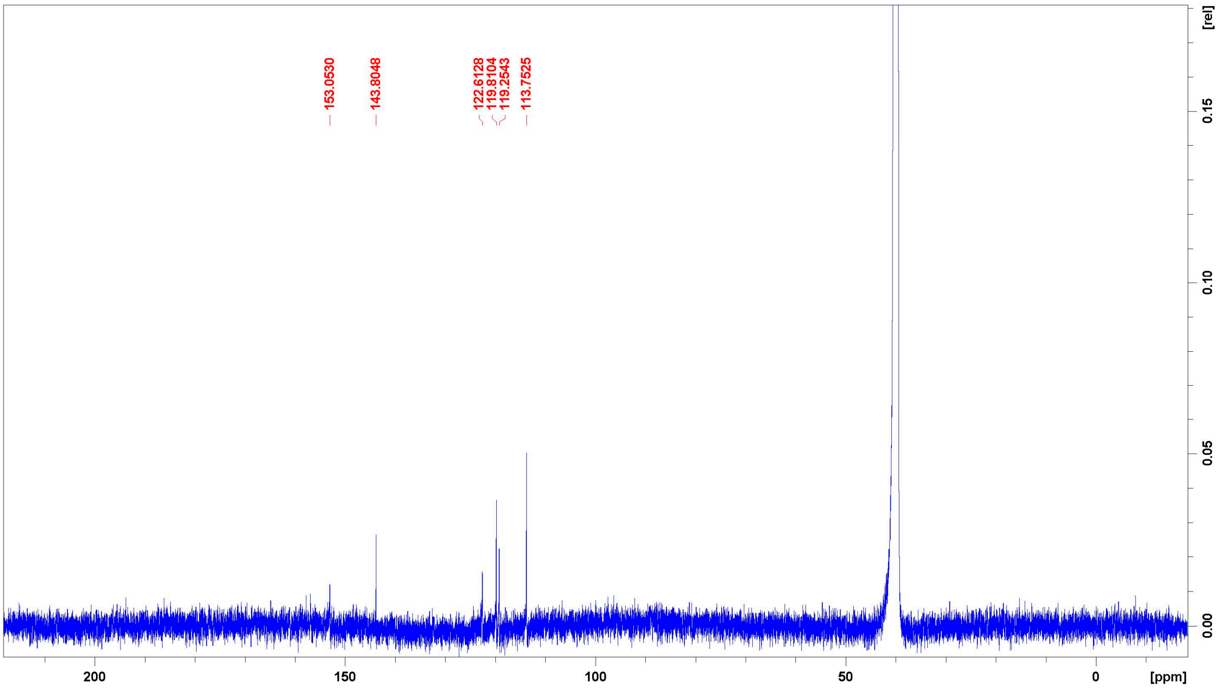

^{13}C NMR (D_6-DMSO, 500 MHz) δ 153.1, 143.8, 122.6, 119.8, 119.3, 113.8.

1.1.12. 2-Acetamido-N-(5-nitrothizol-2-yl)benzamide (6a)

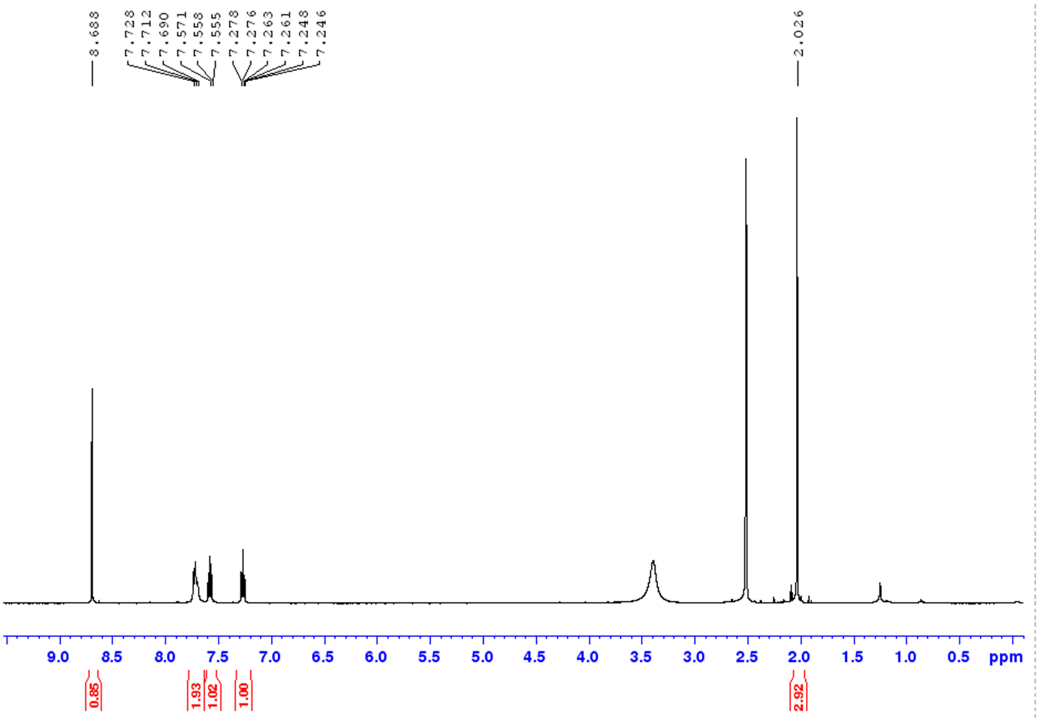

^{1}H NMR (D$_6$-DMSO, 500 MHz): δ 8.69 (1H, s), 7.71 (2H, m), 7.57 (1H, ddd, J = 1.5, 6.8, 8.7 Hz), 7.26 (1H, ddd, J = 1.0, 7.4, 7.7 Hz), 2.03 (3H, s).

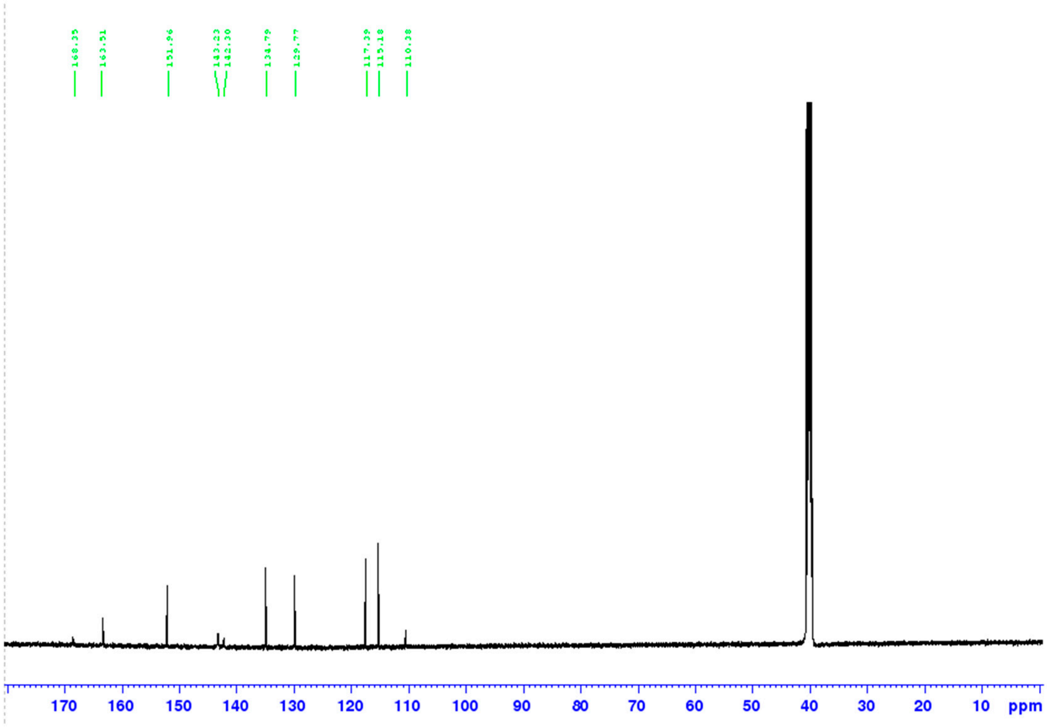

^{13}C NMR (D$_6$-DMSO, 500 MHz) δ 168.3, 163.5, 151.9, 143.2, 142.3, 134.8, 129.8, 117.4, 115.2, 110.4.

1.1.13. 2-Acetamido-5-chloro-N-(5-nitrothizol-2-yl)benzamide (6b)

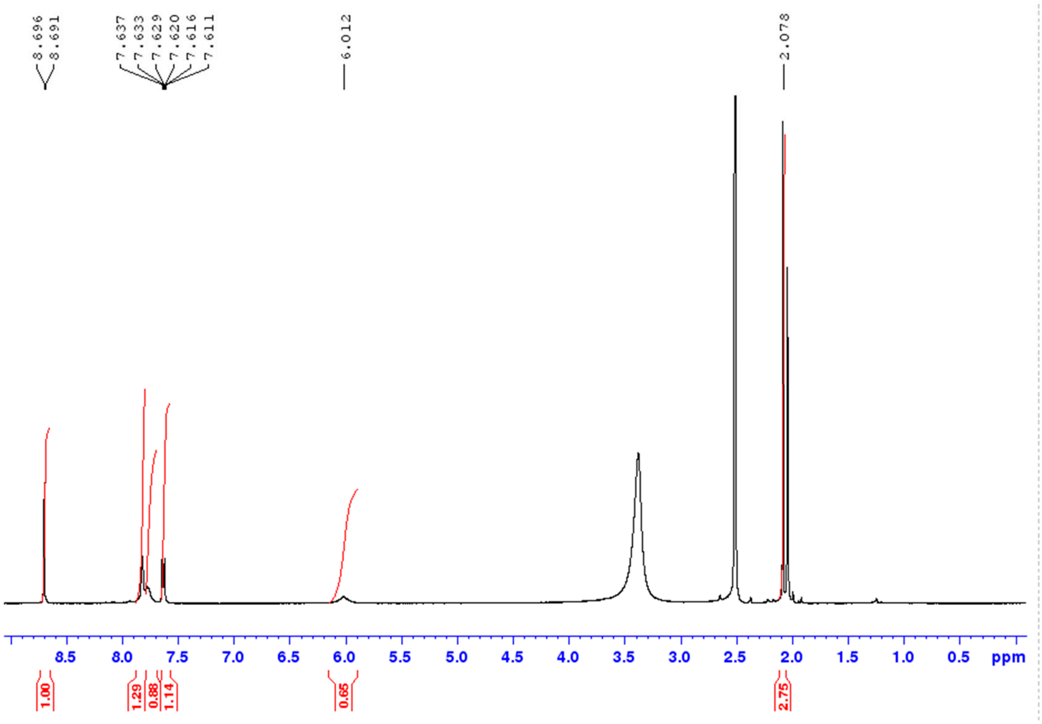

^{1}H NMR (D6-DMSO, 500 MHz): δ 8.69 (1H, s), 7.82 (1H, m), 7.79 (1H, m), 7.61 (1H, dd, *J* = 2.3, 8.7 Hz), 6.01 (1H, s), 2.04 (3H, s).

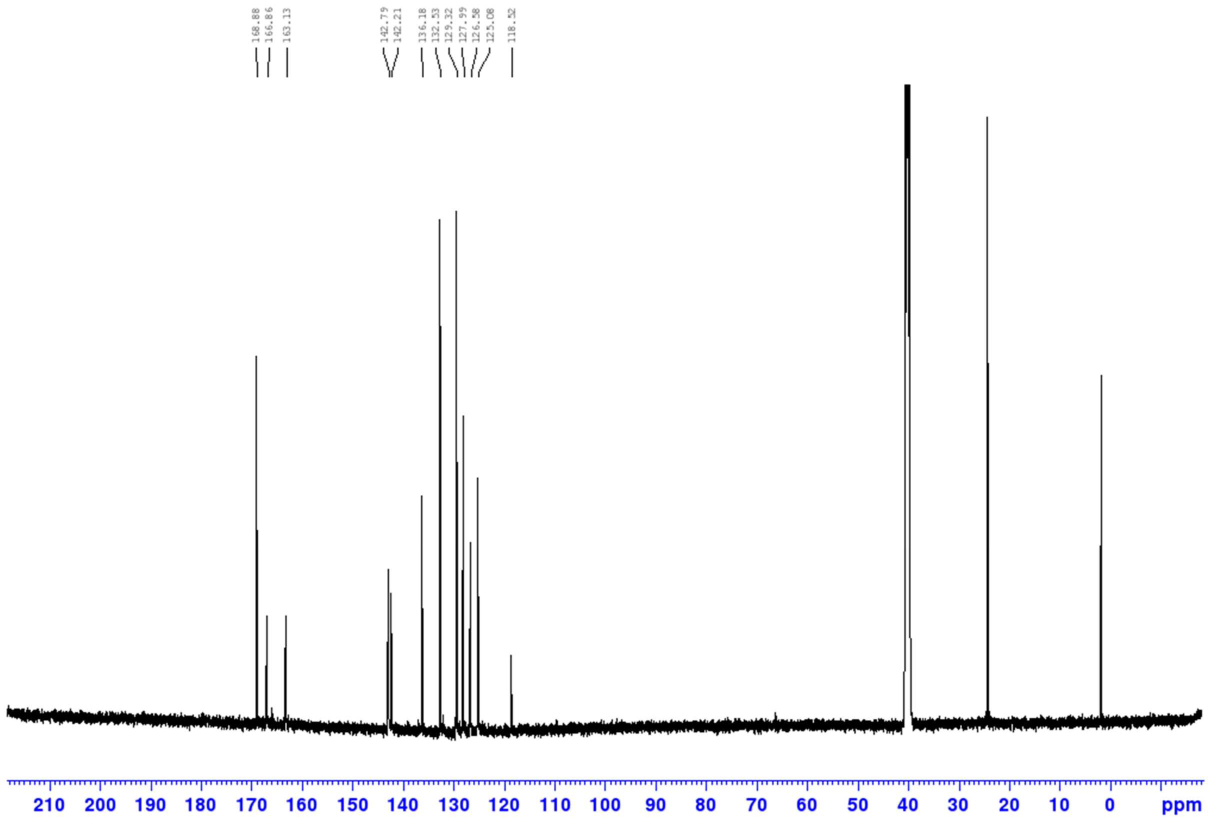

^{13}C NMR (D_6-DMSO, 500 MHz) δ 168.9, 166.9, 142.8, 142.2, 136.2, 132.5, 129.3, 127.9, 126.6, 125.1, 118.5.

1.1.14. 2-Amino-N-(5-nitrothiazol-2-yl)benzamide (7a)

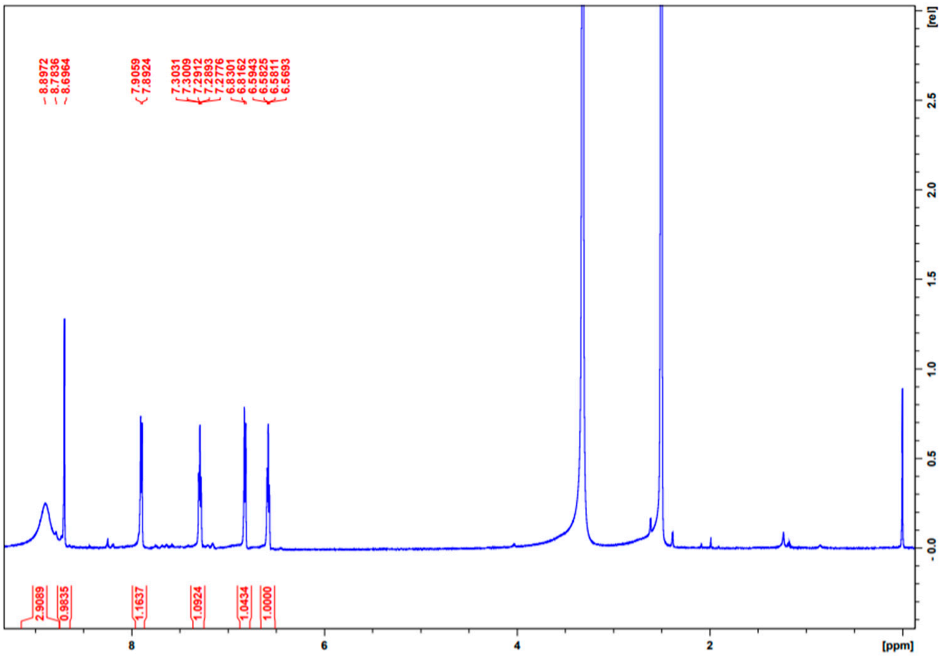

^{1}H NMR (D6-DMSO, 500 MHz): δ 8.89 (2H s), 8.78 (1H, s), 8.69 (1H, S), 7.89 (1H, d, *J* = 8.1 Hz), 7.29 (1H, ddd, *J* = 1.3, 6.8, 7.1 Hz) 6.82 (1H, d, *J* = 8.3 Hz) 6.58 (1H, ddd, *J* = 0.82, 7.1, 7.9 Hz).

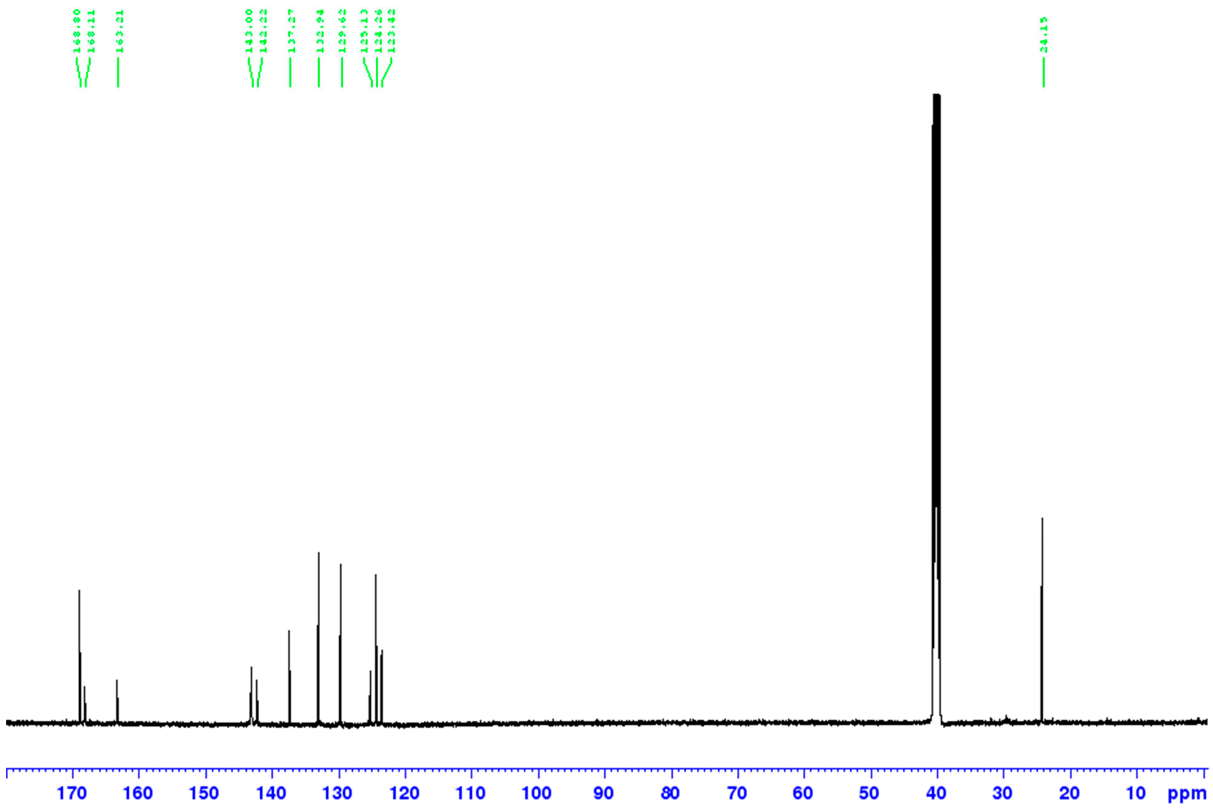

^{13}C NMR (D_6-DMSO, 500 MHz) δ 168.8, 168.1, 163.2, 143.0, 142.2, 137.3, 132.9, 129.6, 125.1, 124.3, 123.4, 24.15.

1.1.15. 2-Amido-5-chloro-N-(5-nitrothizol-2-yl)benzamide (7b)

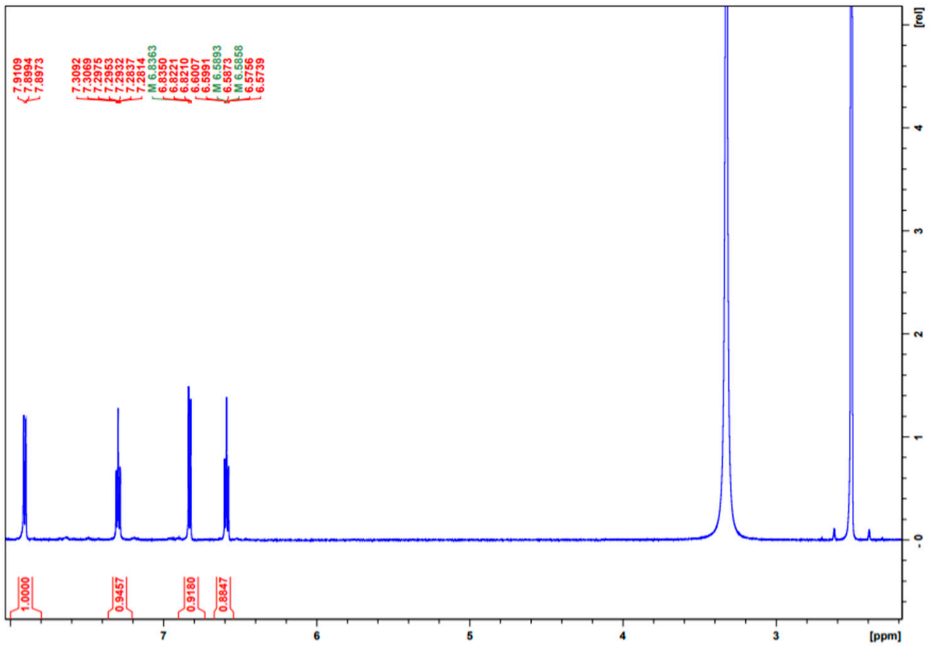

^{1}H NMR (D_6-DMSO, 500 MHz): *δ* 7.91 (1H, dd, *J* = 2.3, 8.1 Hz), 7.29 (1H, m), 6.82 (1H, dd, *J* = 0.8, 8.5 Hz), 6.59 (1H, m).

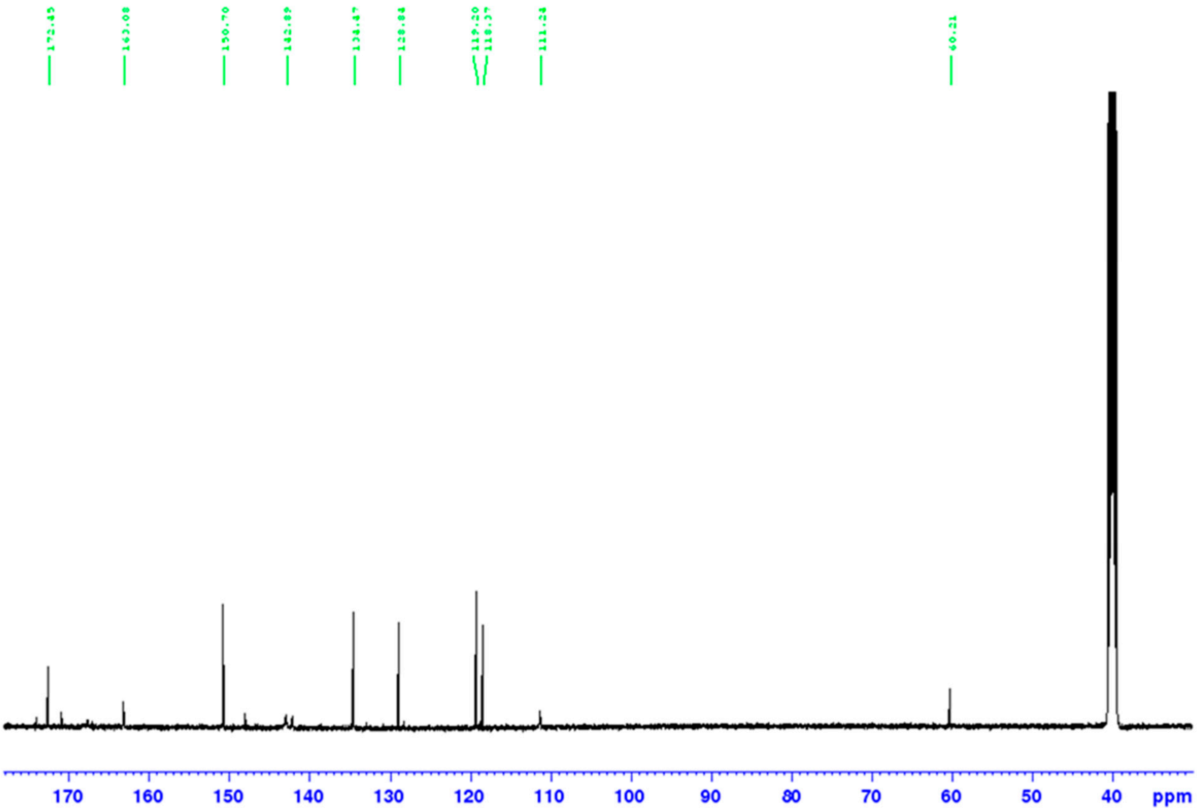

^{13}C NMR (D_6-DMSO, 500 MHz) δ 172.5, 163.1, 150.7, 142.1, 134.5, 128.8, 119.2, 118.4, 111.2, 60.2.

1.1.16. 4-Acetoxy-3-methoxy-N-(5-nitrofuran-2-carbonyl)benzhydrazide (9a)

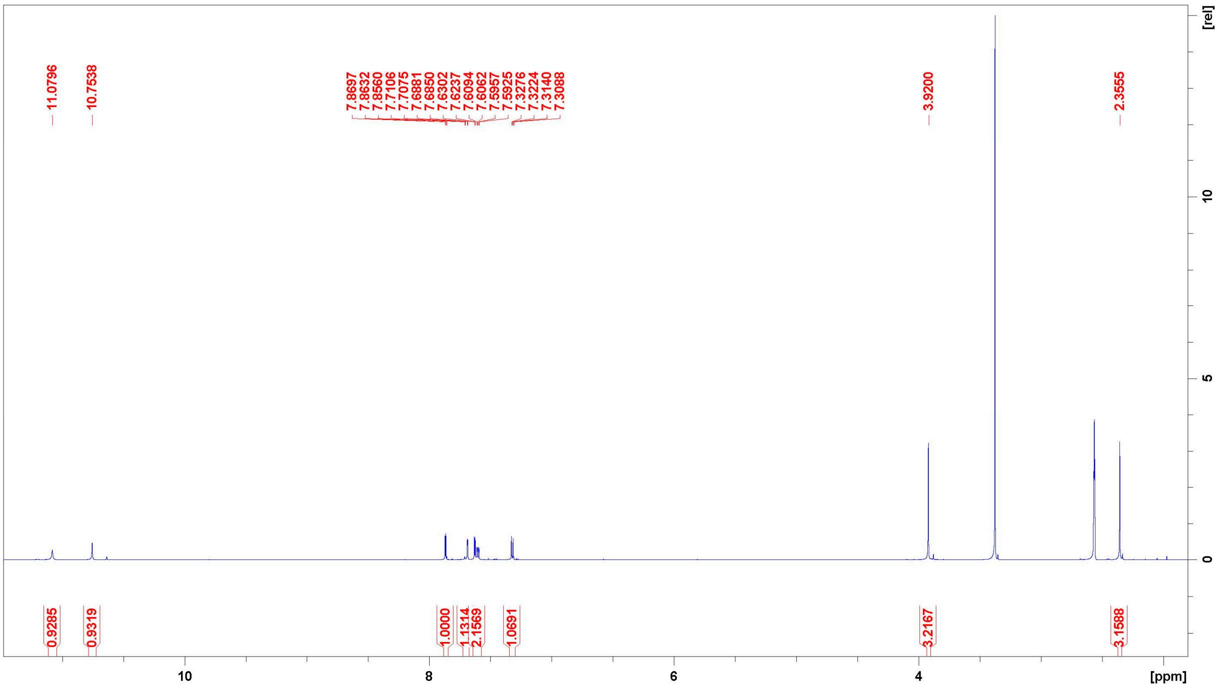

^{1}H NMR ($CDCl_3$, 500 MHz): δ 11.08 (1H, s), 10.75 (1H, s), 7.86 (1H, d, *J* = 3.91 Hz), 7.68 (1H, d, *J* = 1.84 Hz) 7.62 (1H, d, *J* = 3.91 Hz) 7.60 (1H, dd, *J* = 1.9, 8.2 Hz), 7.32 (1H, d, *J* = 8.1 Hz), 3.92(3H, s), 2.35 (3H, S).

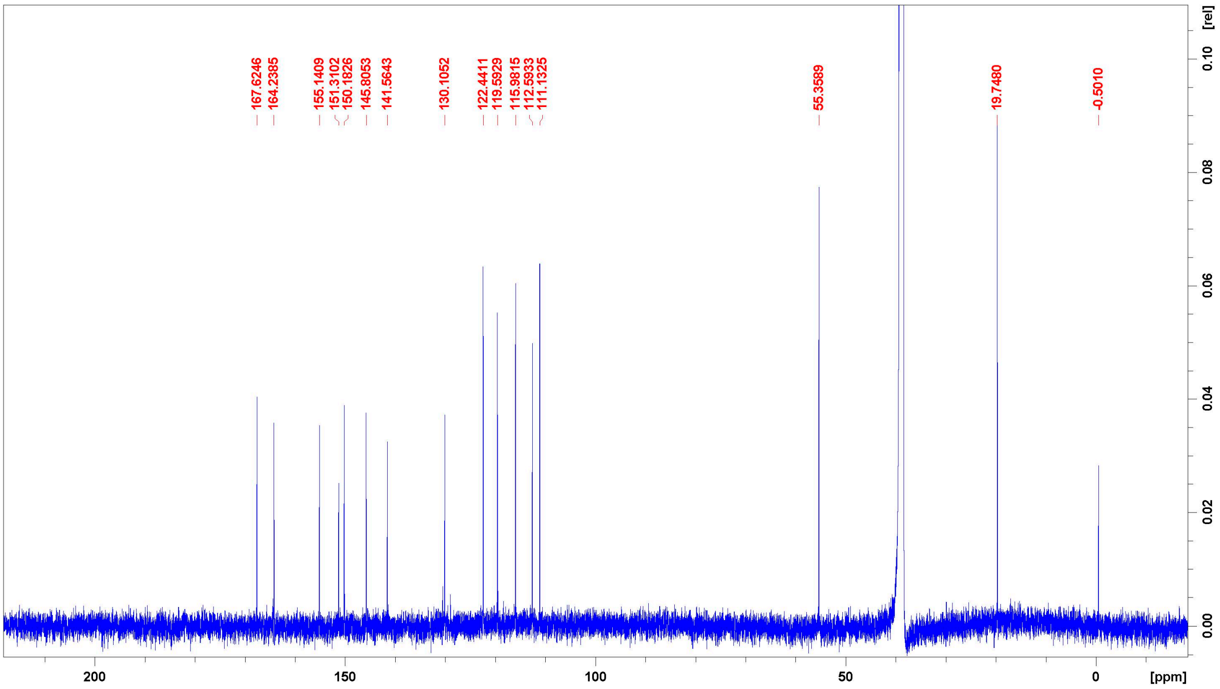

^{13}C NMR ($CDCl_3$, 500 MHz) δ 167.6, 164.23, 155.14, 151.31, 150.2, 145.8, 141.6, 130.1, 122.4, 119.6, 115.9, 112.6, 111.1, 55.4, 19.7.

1.1.17. 4-Acetoxy-3-chloro-N-(2-(5-nitrofuran-2-carbonyl)benzhydrazide (9b)

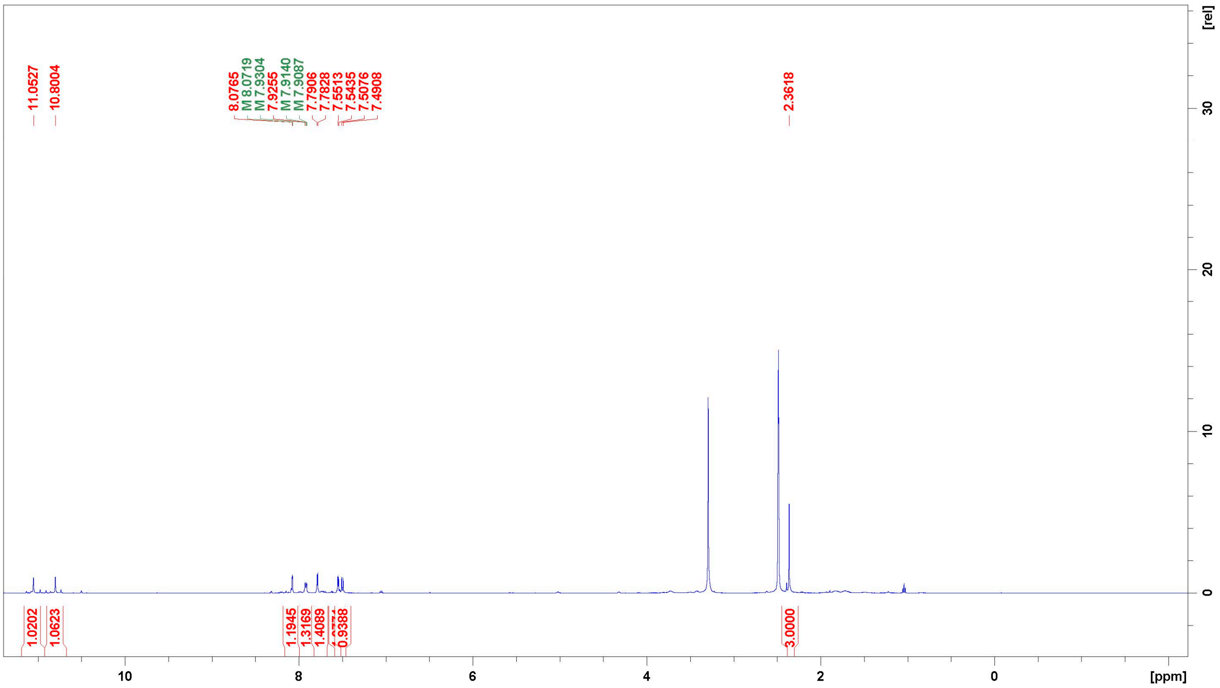

^{1}H NMR (D_6-DMSO, 500 MHz): δ 11.05 (1H, s), 10.80 (1H, s), 8.08 (1H, d, J = 2.3 Hz), 7.91 (1H, dd, J = 2.5, 8.2 Hz), 7.78 (1H, d, J = 3.9 Hz), 7.55 (1H, d, J = 3.9 Hz), 7.50 (1H, d, J = 8.4 Hz), 2.36 (3H, s).

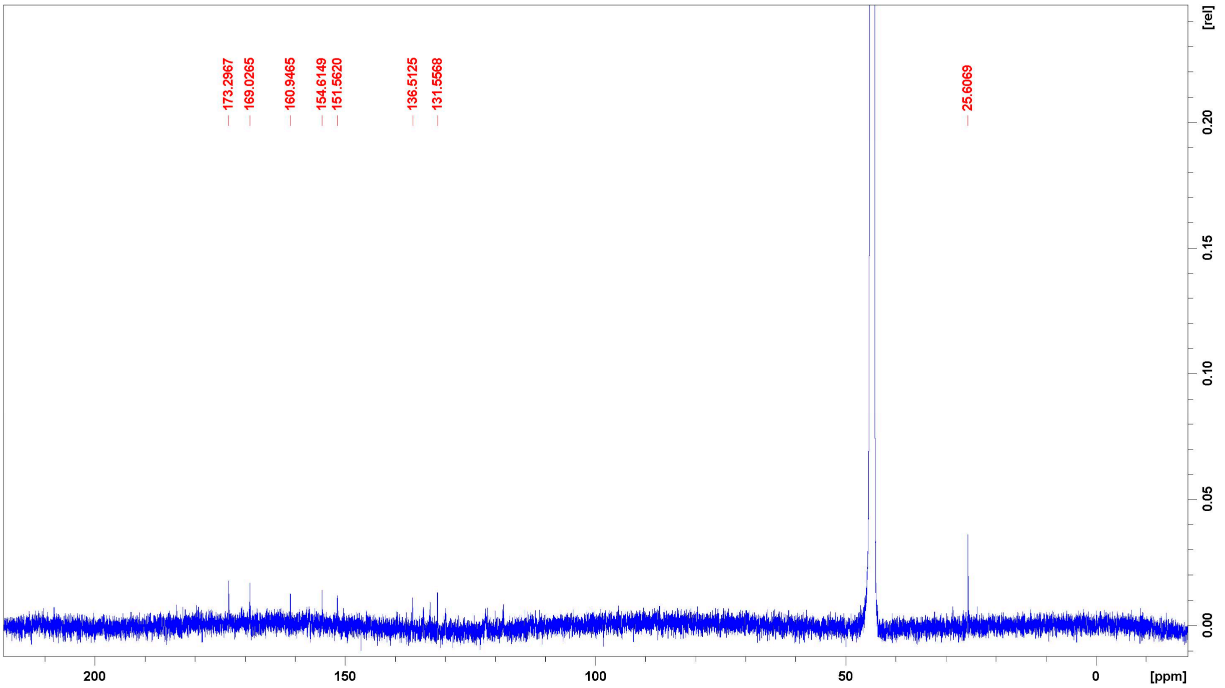

^{13}C NMR (D_6-DMSO, 500 MHz) δ 173.3 169.0, 160.9, 154.6, 151.6, 136.5, 131.6, 25.6.

1.1.18. 2-Acetoxy-5-methoxy-N-(5-nitrofuran-2-carbonyl)benzhydrazide (10a)

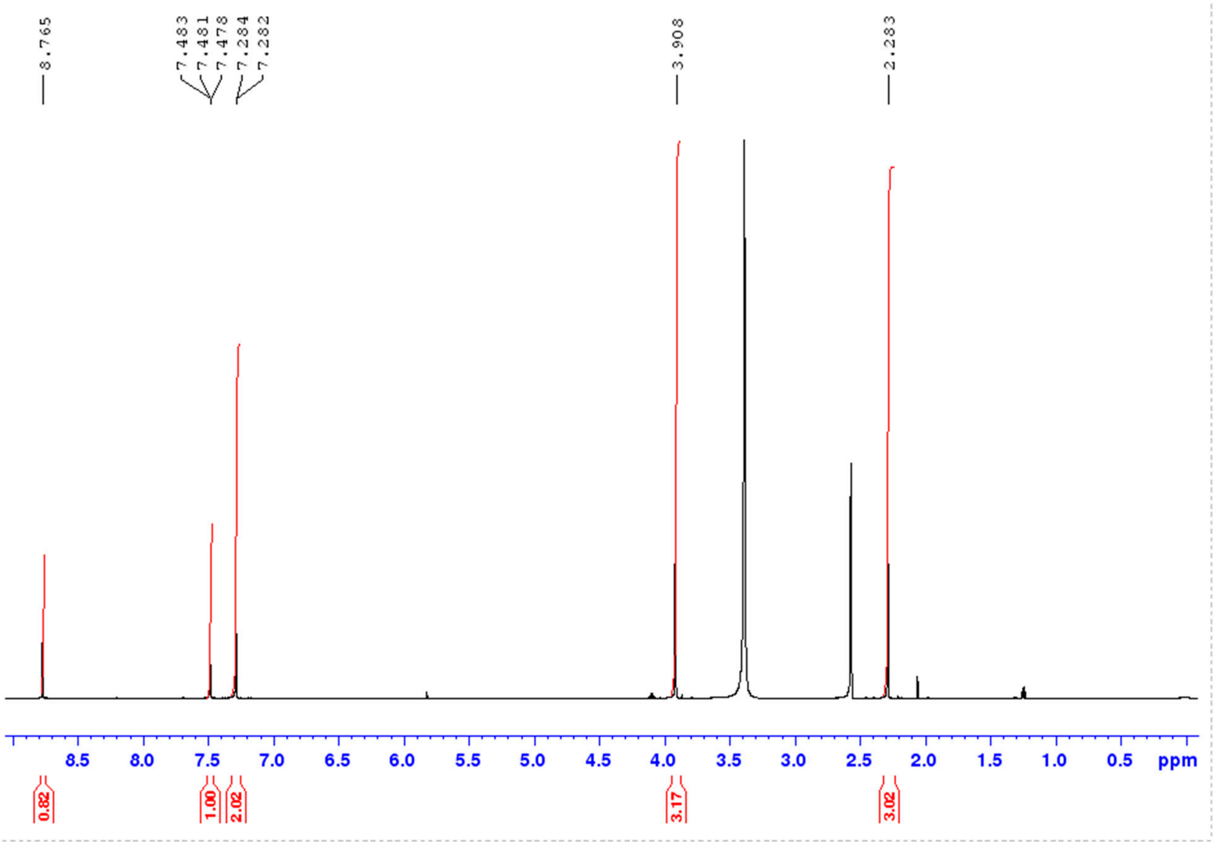

^{1}H NMR (D_6-DMSO, 500 MHz): 8.77 (1H, s), 7.43 (1H, m), 7.28 (2H, m), , 3.91 (3H, s), 2.29 (3H, s).

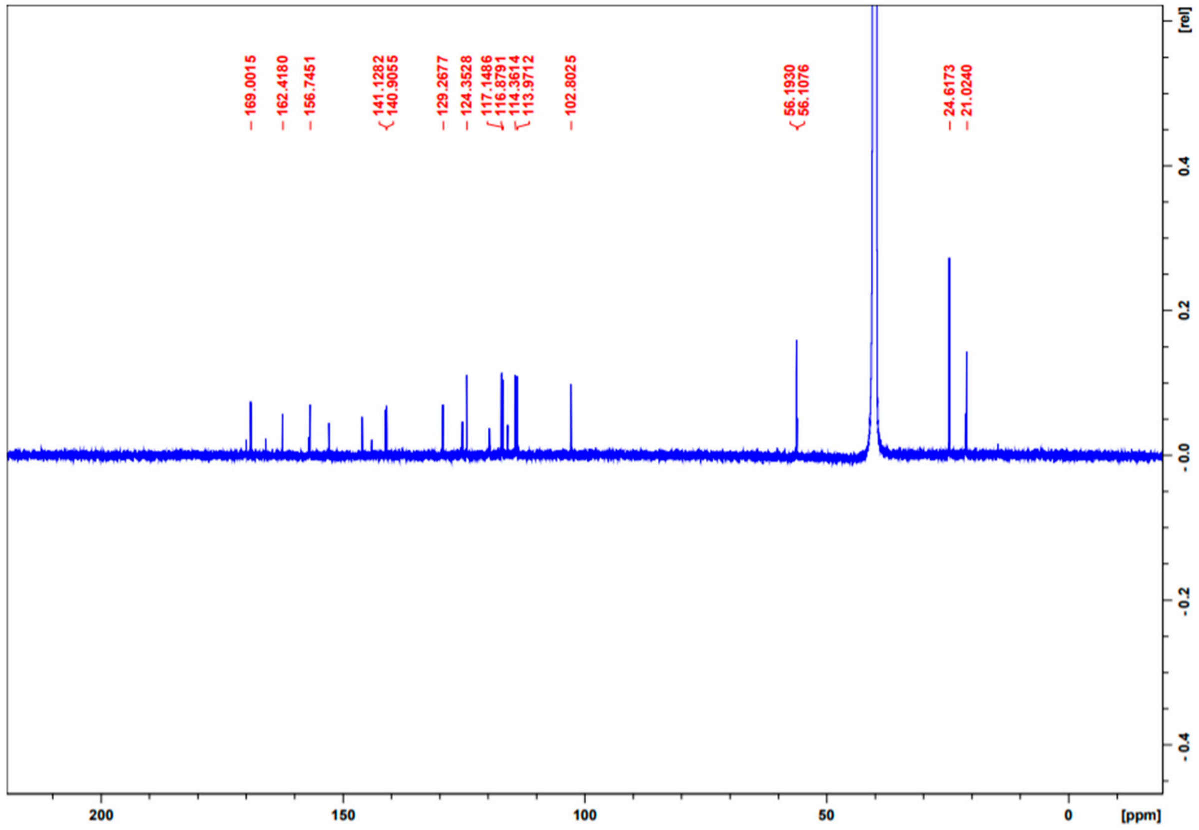

^{13}C NMR (D_6-DMSO, 500 MHz): 169.0, 162.4, 156.7, 141.1, 140.9, 129.3, 124.3, 117.14, 116.8, 113.9, 102.8, 56.2, 56.1, 24.6, 21.0.

1.1.19. 2-Acetoxy-5-chloro-N-(5-nitrofuran-2-carbonyl)benzhydrazide (10b)

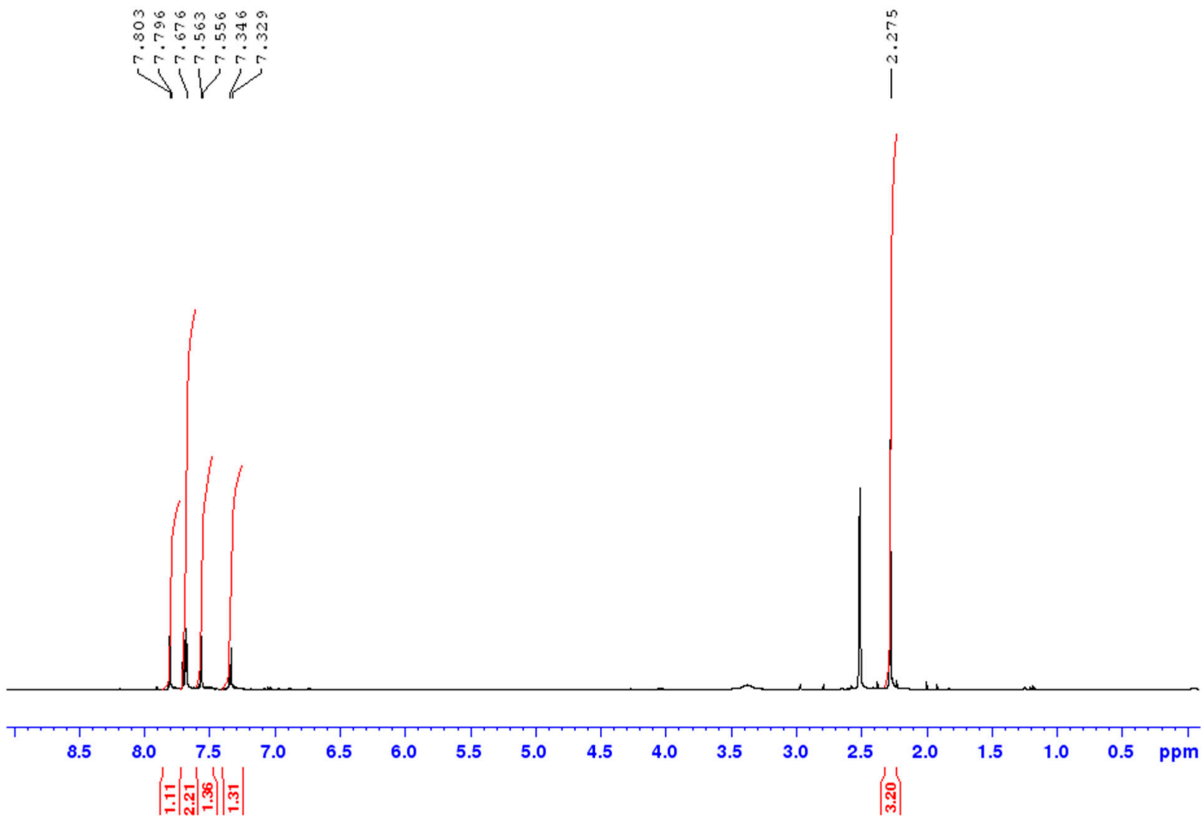

^{1}H NMR (D6-DMSO, 500 MHz): δ 7.80 (1H, d, *J* = 3.9, 7.8 Hz), 7.69 (1H, dd, *J* = 2.6, 5.9 Hz), 7.67 (1H, dd, *J* = 2.4, 3.1 Hz), 7.55 (1H, d *J* = 3.9 Hz), 7.33 (1H, d, *J* = 8.5 Hz), 2.27 (3H, s).

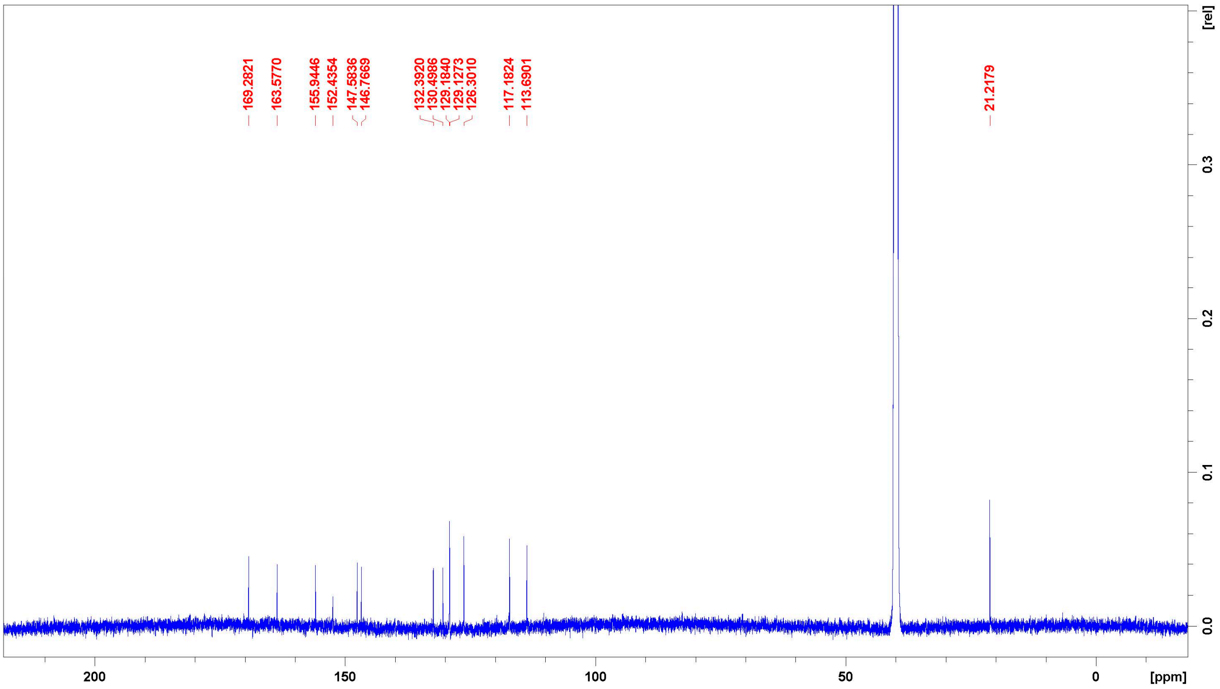

^{13}C NMR (D_6-DMSO, 500 MHz) δ 169.3, 163.6, 155.9, 152.4, 147.6, 146.7, 132.4, 130.5, 129.2, 129.1, 126.3, 117.2, 113.7, 21.2.
